# Gene expression signatures of individual ductal carcinoma in situ lesions identify processes and biomarkers associated with progression towards invasive ductal carcinoma

**DOI:** 10.1038/s41467-022-30573-4

**Published:** 2022-06-13

**Authors:** Clare A. Rebbeck, Jian Xian, Susanne Bornelöv, Joseph Geradts, Amy Hobeika, Heather Geiger, Jose Franco Alvarez, Elena Rozhkova, Ashley Nicholls, Nicolas Robine, Herbert K. Lyerly, Gregory J. Hannon

**Affiliations:** 1 Cancer Research UK Cambridge Institute, University of Cambridge, Cambridge, UK; 2 Department of Pathology & Laboratory Medicine, East Carolina University Brody School of Medicine, Greenville, NC USA; 3 Department of Surgery, Duke University Medical Center, Durham, NC USA; 4 New York Genome Center, New York, NY USA; 5 Department of Dermatology, Boston University School of Medicine, Boston, MA USA

**Keywords:** Breast cancer, Cancer genomics, Transcriptomics, Tumour biomarkers

## Abstract

Ductal carcinoma in situ (DCIS) is considered a non-invasive precursor to breast cancer, and although associated with an increased risk of developing invasive disease, many women with DCIS will never progress beyond their in situ diagnosis. The path from normal duct to invasive ductal carcinoma (IDC) is not well understood, and efforts to do so are hampered by the substantial heterogeneity that exists between patients, and even within patients. Here we show gene expression analysis from > 2,000 individually micro-dissected ductal lesions representing 145 patients. Combining all samples into one continuous trajectory we show there is a progressive loss in basal layer integrity heading towards IDC, coupled with two epithelial to mesenchymal transitions, one early and a second coinciding with the convergence of DCIS and IDC expression profiles. We identify early processes and potential biomarkers, including *CAMK2N1*, *MNX1*, *ADCY5*, *HOXC11* and *ANKRD22*, whose reduced expression is associated with the progression of DCIS to invasive breast cancer.

## Abstract (web summary)

Progression from ductal carcinoma in situ (DCIS) to invasive ductal carcinoma (IDC) remains poorly understood. Here, the authors analyse over 2700 micro-dissected samples using transcriptomics to identify genes that characterise different stages of DCIS to IDC progression, and identify IDC-associated markers within early-stage lesions.

## Introduction

Ductal carcinoma in situ (DCIS) is considered to be a non-invasive precursor to breast cancer, and when found is associated with an ~10-fold increased risk of developing an invasive carcinoma^1^. However, over half of untreated DCIS patients may never develop breast cancer^2,3^. Despite this, conventional treatment typically comprises either mastectomy or breast-conserving surgery coupled with radiation. In order to treat women most effectively and reduce unnecessary treatment, it is vital that we understand more about DCIS and what factors influence the risk of progression to invasive disease. At present, the path from normal ductal epithelium to invasive ductal carcinoma (IDC) remains poorly understood. Current thinking suggests that there is a step-wise progression from a normal duct, through atypical ductal hyperplasia (ADH), to DCIS followed by microinvasion from the duct to established invasive ductal carcinoma. While the ductal epithelium is typically comprised of a mixture of luminal and basal-like cells, ADH and DCIS are expansions of the luminal compartment^4^, with the presence of nuclear and/or architectural atypia. Distinguishing DCIS from ADH is one of the most difficult challenges in breast pathology, and there is marked inter-observer variability, suggesting that not all disease states are easily categorised by morphology alone. In addition, distinguishing low-risk from high-risk DCIS lesions can be difficult at best. A number of studies have examined transcriptional differences between normal ductal tissue, ADH, DCIS, and IDC^5–8^, however, there has been little agreement surrounding genes that mark transitions between tissue states, and studies have often been limited by patient number and tissue quality.

Here we describe the analyses of a large-scale transcriptomic study of over 2700 pathologically annotated and individually micro-dissected regions from 145 fresh-frozen patient biopsies. Focusing largely on DCIS, we combined 1624 RNA-seq libraries from DCIS with 394 libraries from IDC, 258 from atypical ductal lesions, 237 from benign ductal lesions and a further 211 libraries from normal mammary epithelium. Using this data, we were able to describe the evolution of tissue states from the transcriptional changes characteristic of very early lesions, through progression toward, and development of invasive carcinoma. This progression of disease, defined using a fitted principal curve, revealed processes characteristic of different points along the path from normal epithelium to IDC. Considering both Pure DCIS (where no IDC was found in that patient - median clinical follow up 9.6 years) and DCIS from patients diagnosed with co-occurring IDC, we saw that the position of individual lesions on the fitted principal curve was not dictated solely by patient diagnosis. Even among lesions derived from patients having only DCIS, there existed a range of developmental stages, that mirrored those seen in patients that progressed to IDC. We also found that position on the fitted principal curve continuum was not determined by ER/PR or Her2 status, similar to a prior finding detailing a trajectory of changes surrounding tumour stroma^9^, thus potentially indicating that early stage disease results from changes in the same core processes for both of ER+ and ER− negative lesions.

## Results

### The cohort

145 Frozen tissue biopsies, kindly donated to Duke University, were microdissected for DCIS, IDC and other ductal regions of interest (see Methods section for further details). Each individual lesion was carried through for RNA sequencing and quality control checks as described in the methods.

We found that 68% of patients had DCIS mRNA expression patterns that matched their clinical scoring for oestrogen receptor (ER/ *ESR1*), progesterone receptor (PR/ *PGR*), and human epidermal growth factor receptor 2 (Her2/ *ERBB2*) (Supplementary Data 1). Of the 44 patients (32%) that did not, 6 showed a clear difference in ER status, 29 showed a clear difference in PR status and 8 showed a clear difference in Her2 status (where Her2 had been clinically scored). It must be acknowledged however, that where IDC was found in the clinical diagnostic biopsy, it is the IDC that was scored for these markers and not the DCIS, and scoring is based on a number of factors, such as the percentage of invasive tumour cells with nuclear staining as well as the average staining intensity. Within the IDC samples, we also found that 68% of patients matched their clinical scoring for ER, PR and Her2, and the remaining 32% (13 patients) showed distinct deviations in their RNA expression from that of the clinical scoring. Four patients had a clear difference in RNA expression signatures for *ESR1*, *PGR* and *ERBB2*, between their DCIS and IDC. These findings are consistent with the well-established heterogeneity within this disease, and in some cases we found that different DCIS samples scored differently even within the same tissue section, most often for *PGR*.

### Triple-negative DCIS cluster separately

**Fig. 1:**
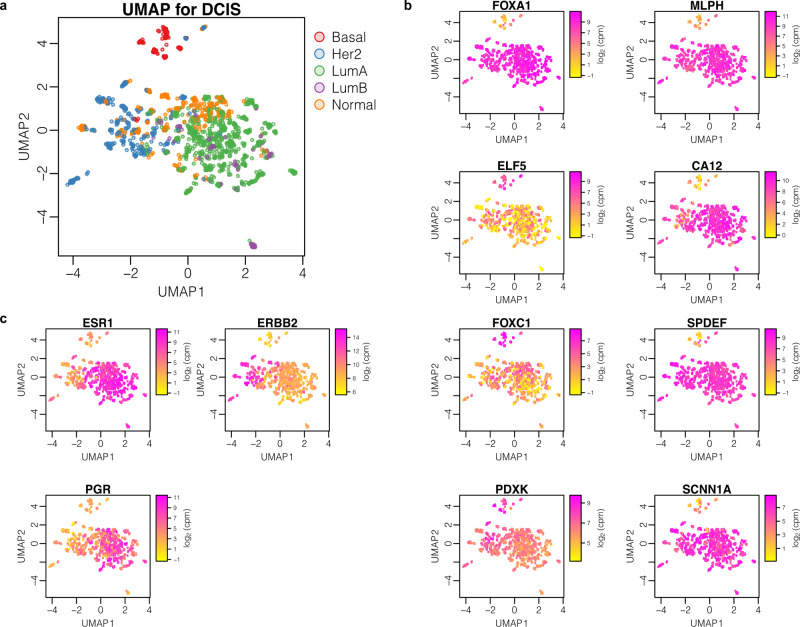
Triple negative DCIS has a transcriptome distinct from other DCIS subtypes. Uniform Manifold Approximation and Projection (UMAP) plots illustrating expression patterns in log_2_ counts per million (CPM), for 1414 DCIS samples by **a** AIMS (Absolute Intrinsic Molecular Subtyping), **b** *ESR1*/*PGR*/*ERBB2* gene expression, and **c** expression of genes that correlate with triple-negative status in DCIS.

To assess whether there were any distinct groups of DCIS samples, we carried out Principal component analysis (PCA) followed by uniform manifold approximation and projection (UMAP) using only DCIS samples (Fig. 1a). This revealed that the majority of samples largely group together, however Basal-like (as defined by AIMS^10^) triple negative (TN) DCIS samples, with low expression for *ESR1* (ER), *PGR* (PR) and *ERBB2* (Her2) (Fig. 1b), form a distinct cluster away from other DCIS samples, including other non-basal-like TN DCIS samples (Fig. 1a and see Fig. S1 for all sample subtype classifications by patients). This is in line with a recent study looking at DCIS subtypes^11^. Differential expression analysis between this Basal-like TN cluster against the other clusters revealed that the mRNA levels for genes encoding pioneer factor, Forkhead Box A1, (*FOXA1*), and Melanophilin, (*MLPH)* were significantly reduced in this cluster as compared to the other subtypes. Other genes showing a strong association with this group are *CA12* - encoding Carbonic anhydrase 12, *SPDEF, FOXC1* and *ELF5* - encoding transcription factors, *SCNN1A*, -encoding the sodium channel epithelial subunit, and *PDXK*—encoding a pyridoxyl kinase (Fig. 1c). *FOXA1* and*, MLPH* are among other genes annotated as being more highly expressed in luminal cells compared to basal cells, and vice-versa for *ELF5*, in studies of mouse mammary glands^12^.

*FOXA1* has recently been highlighted as a potentially useful marker for triple negative breast cancer^13^, and its expression has been suggested to act as a repressor for a subset of basal signature genes^14^. However, a role for FOXA1 as a subtype marker for DCIS has previously been dismissed as no correlation could be seen with protein expression and that of ER^15,16^. Here we also observed that *FOXA1* expression does not systematically differ between ER+ and ER- samples, and its reduced expression is only associated with the basal-like TN samples. The substantial overlap between TN-associated markers identified here, and those found by other studies on invasive breast cancer (including *MLPH*, *CA12*, *FOXA1*, *SPDEF*, *FOXC1*), suggest there is a clear distinction of this subtype even at the pre-invasive stage^17–19^.

### Two gene networks dominate expression differences between co-occurring DCIS and early invasive breast cancer

**Fig. 2:**
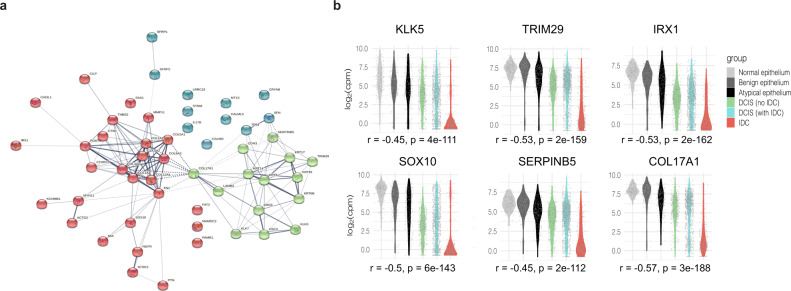
Differentially expressed genes between DCIS and co-occurring IDC. **a** String connectivity with k-means clustering [3 clusters identified by red, green and blue] of the top 53 significant genes. **b** Expression distribution, in log_2_ counts per million (CPM), for example genes that showed a progressive shift among different tissue groups. The Spearman rank correlation (between expression and ordered tissue groups) is given as r = rho. Two-sided *p*-values without correction for multiple testing are indicated.

We sought to leverage our extensive datasets to identify transcriptional differences between DCIS and co-occurring IDC. This would act as a starting point in identifying genes that may be related to the progression towards IDC. We compared the two tissue types from DCIS+ IDC patients, (only for those where we had useable data for both tissue types within a patient), *N* = 33. Using this criterion, we aimed to compare samples that were most closely matched to minimise inherent inter-patient variability. We carried out differential expression analysis between the two tissue groups and found 401 significantly differentially expressed genes (DEGs). Taking the 53 genes with an Adj. P value < 0.00001, we used STRING^20^ to examine their connectivity. We were surprised to find that the genes formed two main, highly interconnected networks, with very few unconnected genes (Fig. 2a). Gene Ontology (GO) term analysis on these two networks revealed an enrichment for upregulated (in IDC over DCIS) genes involved in both extracellular matrix (EM) organisation (FDR 2.5E-16, Fold Enrichment; 29) and cell adhesion (FDR 1.8E-6, Fold Enrichment; 7) and down-regulated genes associated with both epidermis development (FDR 5.3E-8, Fold Enrichment; 18) and epithelial development (FDR 1.6E-06, Fold Enrichment; 7). Specific genes included in each cluster network are frequently associated with these processes, such as *FN1* (Fibronectin 1), and the collagen genes (*COL1A2*, *COL1A1*, *COL12A*, *COL3A1* and *COL5A2*). Other genes, such as *MMP11* (matrix metalloproteinase 11), and *POSTN* (Periostin), encode proteins involved in epithelial cell adhesion and migration, and *THBS2*—encoding Thrombospondin 2, a mediator of cell-cell and cell-matrix interactions. The second cluster network includes *DSC3* and *DSG3*, reported to be expressed only in myoepithelial cells within the basal cell layer, *KRT5*, *KRT14*, *KRT6B* and *KRT15*, markers for basal epithelial cells, and *KLK5* and *KLK7*, whose encoded proteins are considered to be involved in desquamation^21^. We noted a substantial overlap between genes in this cluster and those found to be differentially expressed in basal cells (as compared to luminal cells) in mouse and human mammary glands^22,23^. Considered together, these data could suggest that the expression changes we observe in the down-regulated genes may be reflective of a loss in the basal compartment of the duct. Carrying out the same differential analysis on a per subtype basis had limited statistical power for all but the Luminal A subtype, due to the reduced sample sizes (Her2 *N* = 6, LumB *N* = 3, Basal *N* = 3 and Normal Like *N* = 3). However, we did find many of the 53 genes from the combined analysis also ranked highly in the individual subtype analyses, with Basal patients sharing the least (DEGs can found in Supplementary Data 2).

### DCIS progression from normal epithelium to IDC

Given the strong presence of just two dominant processes that appeared to be contributing to the transition from DCIS to IDC, we examined how the integrity of the basal layer and the EM may differ in our other tissue types, or disease statuses (Pure DCIS or Not Pure DCIS). Looking at these same 53 genes we noted in some cases a progressive shift from expression levels in normal ductal tissue to that seen in IDC (Figs. 2b and S2). Interestingly, for some genes, some DCIS samples displayed an expression pattern that was more reflective of normal epithelium while others more closely resembled IDC, even if the samples were isolated from the same patient. This led us to hypothesize that some DCIS samples are more closely related to their normal counterparts and others more related to their invasive counterparts, and that perhaps this indicated a possible continuum of tissue states represented during disease progression, within an individual patient.

**Fig. 3:**
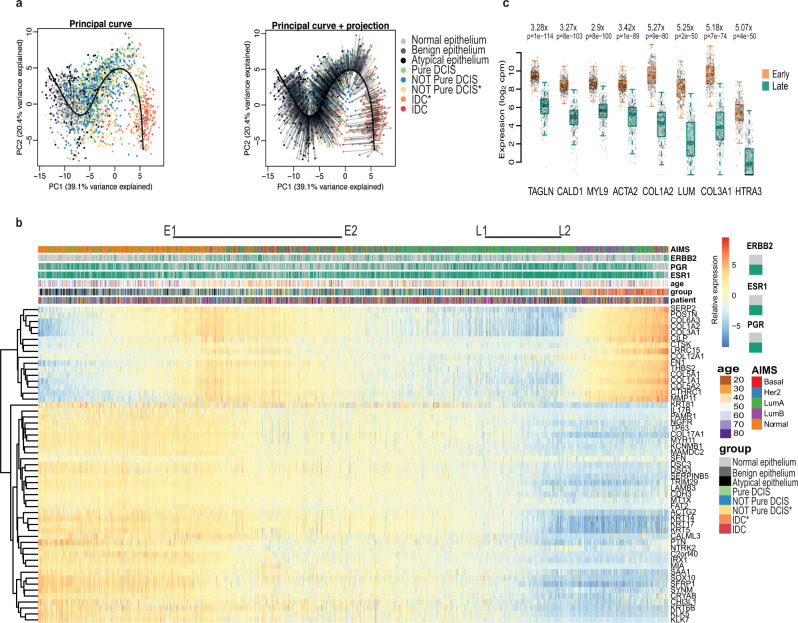
Generating a pseudo-time for DCIS. **a** Principal component analysis (PCA) plot based on the most significant (p < 0.00001) differentially expressed genes between DCIS and co-occurring IDC. All samples plotted according to principal components 1 and 2 (PC1 and PC2 respectively) with their fitted principal curve (left), and with their projection onto the curve (right). **b** Heatmap showing expression of each of the 53 genes with samples ordered by their projection to the principal curve. Top bars indicate AIMS subtype classification, *ERBB2*, *PGR*, and *ESR1* status, age of patient at the time of consent, tissue classification group for each sample, and patient distribution. Relative expression is provided as log_2_ counts per million (CPM) minus the mean log_2_ CPM for each gene. E1 – E2 indicate the Early stage and L1 – L2 indicates the Late stage. The ‘*’ assigned for ‘Yellow Not Pure DCIS’ and ‘Orange IDC’ indicates samples used in the analysis comparing gene expression of DCIS vs IDC for co-occurring patients. ‘Blue Not Pure DCIS’ and ‘Red IDC’ are from tissue biopsies that did not have co-occurring DCIS and IDC in the same sections and were therefore not used for this expression analysis. (**c**) Boxplots illustrating per sample expression data for highly differential genes found when comparing samples in the Early group (E1-E2) with those in the Late group (L1–L2). Differential expression analysis was done using limma-voom and two-sided p-values were adjusted for multiple testing using Benjamini-Hochberg correction. Centre line represents the median, box limits represent upper and lower quartiles, whiskers represent minimum and maximum values and at most 1.5x the interquartile range. Each point represents a sample, *n* = 339 early and *n* = 287 late samples from 106 patients).

To explore this idea, we used these same 53 selected genes that best separated DCIS from IDC (Adj.P < 0.00001) to perform a pseudo-time analysis using a fitted principal curve onto a PCA plot of all our samples (Fig. 3a). We saw that the normal and benign epithelial tissue samples aggregated towards one end of the fitted curve and IDC tissue and DCIS with co-occurring IDC clustered at the other, despite the normal and atypia samples not factoring into the selection of these genes. We then ordered all tissue samples, normal, benign, atypia, DCIS and IDC, by their projection onto the fitted principle curve. We created a heatmap showing expression changes for these genes with sample order matching that from the principal curve projection (PCP) (Fig. 3b). This analysis of early breast cancer seemed to reveal how fundamental processes were associated with progression toward invasive disease. Position along the PCP was independent of ER/PR/Her2 status. Moreover, triple negative samples, despite clustering away from other samples on a UMAP when using all genes (Fig. 1a), or even a UMAP created with just these 53 genes (Fig. S3), did not drive the separation on a PCA. This analysis therefore captured the major expression changes shared across most patients, rather than any particular subtype. We observed a gradual loss of expression for genes involved in the epidermis/epithelial development, as we transition from the more normal-like/early-stage DCIS to the later stage DCIS samples and IDC samples. This suggests a progressive breakdown of epithelial architecture, most likely reflecting a loss of integrity in the basal epithelium.

We carried out XCell analysis^24^ to look for changes in cell type contributions that may occur along this transition, and found further support for epithelial loss with a gradual decline in the enrichment for epithelial cells within each sample when placed in the order of the PCP (Fig. S4).

**Fig. 4:**
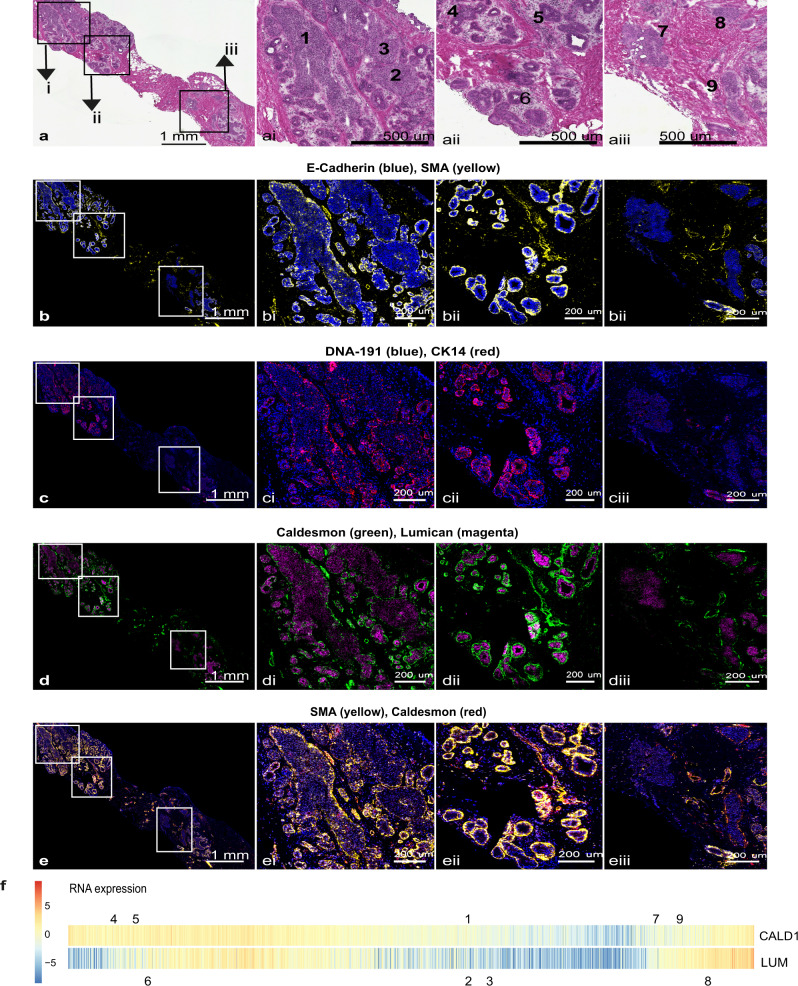
Imaging Mass cytometry of ductal regions from patient sample CBZ (LumA subtype). The single slide was analysed in one continuous scan and magnified regions retained the same intensity threshold. **a** shows H&E stained section, this same section was destained and used for IMC. Boxed areas indicate the corresponding magnified regions shown in ai, ii and iii. **b** shows E-Cadherin and SMA, with corresponding boxed areas for i, ii and iii. **c** Shows CK14 and nuclear stain DNA-191, with corresponding boxed areas for i, ii and iii. **d** shows Caldesmon and Lumican and **e** shows SMA and Caldesmon, with corresponding boxed areas for i, ii and iii. Numbers on the H&E images indicate regions with expression data from adjacent sections. **f** shows the relative gene expression of *Cald1* and *Lum* (as in Fig. 3b) with all samples ordered along the PCP continuum. Numbers above and below pair up with numbers in (a) and mark the position on the PCP continuum for the two (adjacent) data points corresponding to each region.

To understand better the changes that occur just within DCIS as they progress closer to the transcriptomic patterns of IDC, we compared DCIS samples from the early part of the PCP (Fig. 3b, E1-E2) with DCIS samples from the later part of the PCP (Fig. 3b, L1–L2) we found that a number of smooth muscle related genes were down regulated in the later stages with *TAGLN*, *CALD1*, *MYL9* and *ACTA2* being most significant (Fig. 3c and Supplementary Data 2). In addition, along with the Collagen genes *Col1A2* and *Col3A1*, *LUM* – encoding Lumican, a small leucine-rich proteoglycan found to be associated with EMT, invasion and metastasis^25^, and *HTRA3*- encoding High-Temperature requirement Factor A3, were found to have the greatest fold change (Fig. 3c). Changes in the expression of Caldesmon (encoded by *CALD1*), surrounding the mammary duct, have previously been observed, with a recent study showing this protein to be upregulated in the epithelium of mammary ducts in both mice and humans during lactation^26^. To visualise the protein distribution in samples representative of different stages of the PCP we carried out imaging mass cytometry (IMC) (Fig. 4).

As can be seen from the representative slide shown in Fig. 4 – where all IMC images are taken from a single slide, previously stained with Hematoxylin and Eosin (H&E) (Fig. 4a), and imaged in one continuous scan – protein staining for Cald1 (Fig. 4d, e), Smooth Muscle Actin (SMA) (Fig. 4b, e), and to a lesser extent Cytokeratin 14 (CK14) (Fig. 4c) appear to overlap in localisation, and the level of intensity, representative of ion count, is comparative to the relative RNA expression level within the corresponding lesions from the adjacent tissue section (Fig. 4f). Those regions located early in the PCP (4, 5, and 6 – a-e parts ii from Fig. 4) have a relatively intact layer of Cald1-expressing cells surrounding the duct, whereas regions further along the PCP (1, 2, 3 and 7 – a-e parts i and iii from Fig. 4) show a much more broken or absent layer of Cald1-expressing cells. Regions towards the very end of the PCP continuum (8 and 9 – a-e parts iii) are starting to upregulate *Cald1* expression and this can be visualised in areas around the duct. The underlying reason for this observation remains unknown however. It is possible that Cald1 is marking fibroblast cells surrounding the ducts, as this marker is frequently associated with this cell type^27^. Cald1 positive cells located around regions 8 and 9 (Fig. 4e part iii) may be an alternative form of cancer-associated fibroblast (CAF), noted to have a different staining pattern with SMA compared to other regions. This is supported by a recently published study describing a shift in fibroblast phenotype, from normal fibroblasts lining the DCIS ducts, to cancer-associated fibroblasts lining DCIS ducts in patients that later developed IDC^9^. A prior study on glioma neovascularization has also described differential expression of splicing variants of *Cald1* in tumour vessels as compared to normal vessels, resulting in upregulation at the protein expression level within the tumour. This was seen to be correlated with a down regulation of the tight junction proteins occludin and Zo-1 – important regulators of mammary epithelia permeability^28^. As our RNA-seq data was not able to reveal transcript variants, we cannot yet attribute this change in expression towards the later end of the PCP to any particular splice variants. Protein expression for Lumican (Fig. 4d) also appears to follow the trajectory indicated by the PCP, however in this patient, is strongest in the earlier region of the continuum.

Previous studies have suggested a similar breakdown of myoepithelium during the progression towards IDC, using human breast cancer cell lines and a few select markers^29^, and very recently a study of human breast tissue with known markers of myoepithelial cells^9^, lends support to the broader set of expression changes that can be referenced to our PCP.

### The epithelial to mesenchymal transition marks both the early and late stages in progression towards IDC

The PCP revealed a wave in expression of genes relating to the extracellular matrix and cell adhesion, suggestive of a migratory phenotype, initiating relatively early along the continuum and one later, coinciding with the inclusion of the IDC samples (see Fig. 3b). As prior studies^30^ have indicated that multiple DCIS lesions within an individual patient may be of shared origin, one might imagine that an early loss of adhesion might facilitate spread throughout ductal networks, indeed ~40% of patients with a DCIS diagnosis, are found to have multifocal disease^31^, as defined by more than one distinct site of DCIS. Subsequent proliferation and filling of ducts may see a return of cell adhesion with a loss of this property again preceding or coinciding with invasion.

**Fig. 5:**
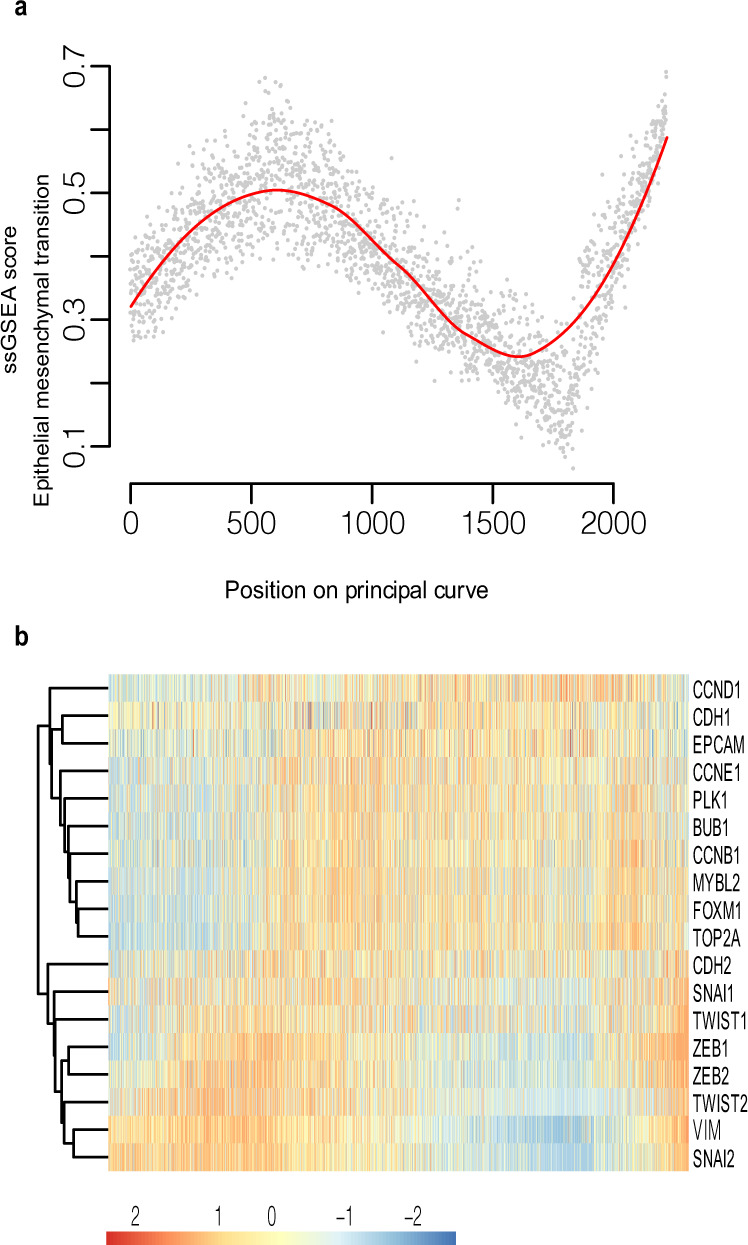
Predominant Hallmark signatures that vary along the PCP continuum. **a** Single-sample Gene Set Enrichment Analysis (ssGSEA) score for the Epithelial to Mesenchymal transition Hallmark signature. Samples are ordered according to the principal curve projection. **b** Heatmap showing expression of key proliferation genes (CCND1 – Top2A) and key EMT (CDH2-SNAI2) genes. Samples were ordered according to the principal curve projection. Relative expression is provided as log_2_ counts per million (CPM) minus the mean log_2_ CPM for each gene.

To gain a greater understanding of processes that could be occurring along the PCP, we applied the entire transcriptome, to the MSigDB Hallmarks database to look for gene set signatures. We found the expression pattern of genes associated with the Epithelial to mesenchymal transition (EMT) hallmark signature to closely mirror many of those genes used to generate the PCP (Figs. 5a and S5), and also reflected the position of the principal curve along PC2 in our PCA (see Fig. 3a). It has long been proposed that cells within DCIS lesions undergo an EMT along their path toward invasiveness, however, the ability to position our samples along a disease trajectory has allowed us to detect that EMT not only occurs in DCIS samples positioned at the transition to invasive disease, but also at a second time, much earlier in the disease continuum, when the epithelial architecture surrounding the duct presumably remains intact (region E1 to E2 of Fig. 3b).

The emergence of EMT at very early time points in disease could suggest that cells require this process to migrate though the ductal system, disseminating and forming multifocal DCIS. Following an early dissemination phase, cells may again adopt a more epithelial character as they become proliferative, with a later acquisition of mesenchymal features coinciding with exit of tumour cells from the duct. The possibility of both an early and late EMT phase could be something to consider when using EMT markers to group DCIS cells into those that may be pre-invasive versus more indolent. EMT potentially occurring twice during the progression from normal epithelium to IDC might suggest that it is alone insufficient to enable invasion but that it must be coupled to breakdown of the myoepithelium for transformed cells to escape from the confines of the duct.

### Cell proliferation increases after the early EMT phase

We also identified additional processes within the MSigDB Hallmarks database that were not represented by our 53 PCP genes, yet still correlated with changes in tissue states along the path between normal epithelium and invasive disease. We observed what appeared to be an altered regulation of the G2/M checkpoint signature in the early stages of the PCP (Fig. S7), however only a subset of genes were actually contributing to the signal. On closer examination we found that these genes were all associated with proliferation, including genes identified as being key to the proliferation signature (*MYBL2*, *BUB1* and *PLK1*)^32^. This increase in expression of proliferation genes appears to initiate just after the first peak in expression of EMT related genes (Fig. 5b), supporting the notion that after migration through the ducts, cells resettle and proliferate as they occupy new sites.

### Reduced expression of *GLTSCR2* and perturbation of ribosomal biogenesis is an early DCIS event

As we generated what appeared to be a progression of ductal transformation, we next sought to identify genes that may be altered during the earliest stages of disease initiation. For this we focused first on the DEGs between all normal (hereafter normal refers to the non-neoplastic normal and benign tissues) tissue samples and all Pure DCIS (with the notion that Pure DCIS samples are less likely to be influenced by the transcriptional changes that come with the presence of IDC). We then looked for shared genes also significant between normal and DCIS, only using samples in the very early part of the PCP (prior to E1 in Fig. 3). In doing this we retained the added strength of a large data set by using all samples but removed the strong expression signature that arose from the onset of increased proliferation and EMT (that came after E1 in Fig. 3). We found *GLTSCR2*, also known as *PICT-1*, to be the most significant DEG when using all normal and all Pure DCIS samples (FC; 1.7 Adj. P; 2.8e-69) and more highly expressed in the normal tissue samples (Fig. S6). This was also one of most significant DEGs in the very early PCP samples (FC; 0.9, Adj. P; 1.6e-14). GLTSCR2, is thought to act as a tumour suppressor^33,34^ and has been shown to translocate to the nucleoplasm, provoked by ribosomal stress, where it interacts with, and stabilizes p53 to inhibit cell cycle progression^35^. Decreased expression was seen to delay DNA repair and abolish G2/M checkpoint activation^33^. The genes *RPL5* and *RPS6*, encoding ribosomal proteins, are, after *GLTSCR2*, the most significantly down regulated genes when comparing all Pure DCIS samples with all normal ductal tissue, (FC; 1.3e-66 and 1.1e-57 respectively), and both genes were also among the most significant DEGs when comparing samples from very early in the PCP continuum. In addition to their role in the ribosome, RPL5 and RPS6 have been shown to be essential for the activation of p53 in response to DNA damage^36^. Pairing the top 100 DEGs between all Pure DCIS and all normal samples, with highly significant DEGs (Adj. P < 1e-10) from the same comparison using only the very early samples, we found 44 overlapping genes, with 19 of these related to ribosomal biogenesis (Table S1). Although ribosomal proteins appear to function in a variety of different ways, there is increasing evidence for their role in tumour development^37,38^, and it is possible that what we are observing at the early stages of the PCP could reflect their involvement in the initiation of DCIS. In addition to ribosomal-related genes, we also observed a significant down regulation in DCIS samples for *NFIB*, encoding the transcription factor Nuclear Factor I B, with this gene being the most significant DEG when comparing very early DCIS with normal epithelium samples (FC; 1.3e-28) (Fig. S6). *NFIB* is part of the NFI gene complex, together with *NFIA*, *NFIC* and *NFIX*. Recent work has described Nfic as being a regulator of ribosomal genes within the pancreas^39^. However, the ribosomal genes affected by Nfic share very little in common with the genes we find most differential in our analysis, and as yet no other work has associated *NFIB* with modified expression of ribosomal genes, thus the expression changes here could be reflective of an additional process in early disease. Current understanding of the transcription factor, Nfib, in breast cancer associates the over expression of this gene with metastasis^40,41^, however, it has also been demonstrated using a prostatic mouse model that heterozygous and homozygous loss of Nfib can lead to epithelial hyperplasia^42^. RNA-seq analysis from this same study, comparing *NFIB* ^-/-^ to *NFIB* ^+/+^ prostatic grafts, identified 138 DEGs, some of which, such as *FOXC1* and *SOX10*, are also differential in our analyses of both early PCP samples, and in all normal vs DCIS samples, suggesting a shared role for Nfib in both prostate and mammary epithelial tissue.

### DCIS progression follows divergent paths depending on hormonal status

In contrast to the early stages of our PCP continuum, we see a divergence in dominant hallmark signatures later in the PCP when we look at samples grouped by oestrogen receptor status (Fig. S7). Not surprising the Oestrogen Response signatures are up in ER+ samples as they progress closer to IDC, and this is not observed in ER- samples. The later stage of the PCP for ER- samples appears to engage an immune response as reflected by a substantial rise in both the Interferon Gamma and Interferon Alpha response signatures. We also see a reduction in the Oxidative Phosphorylation signature in ER- samples.

### Potential indicators of progression competence within early-stage lesions

The ability to discriminate DCIS lesions that have a higher potential to progress to invasive disease would have enormous impact in the clinic. We therefore asked whether we could identify indicators of progression potential that could be used even if a patient presented with DCIS and no evidence of invasive disease. The position of a patient’s DCIS sample along the PCP did not appear to be indicative of that patients’ diagnosis, i.e. Pure DCIS or IDC (mean difference in position on the PCP between Pure DCIS and Not Pure DCIS – 130; *p* = 0.11; Welch’s two sample t-test), this is in contrast to a recent study, describing Pure DCIS patients as having a less intact myoepithelium as compared to those that later developed IDC^9^, indeed we did not see an enrichment for Pure DCIS patients in the later end of our PCP where samples display reduced expression for epithelial related genes^43^. Having the transcriptomes of micro-dissected lesions ordered along a continuum however, offers the opportunity to probe a more comprehensive dataset with unbiased markers. Given that our PCP indicates a distribution of DCIS expression phenotypes, we examined DCIS samples from three groups: those early in the PCP – defined by being closer to the normal samples (region E1 to E2, Fig. 3b), in the middle of the PCP (between E2 and L1) and late in the PCP, adjacent to the IDC-enriched region (region L1 to L2). Comparing the transcriptome of Pure DCIS to Not Pure DCIS revealed 308 DEGs for samples within the early part of the PCP, 206 for the mid region, and just 90 for the late stage of the PCP. The difference in the number of DEGs as we progress along the continuum supports our ordering of samples and suggests that the distinction between samples derived from Pure DCIS patients and patients where DCIS is associated with invasive disease becomes less apparent as the disease progresses along the PCP. This might be expected if lesions are converging on a phenotype similar to that of invasive disease. Interestingly, comparing DCIS samples taken from the very end of the PCP (past L2) with IDC samples from the same region, found no consistent DEGs suggesting these very late DCIS samples are indistinguishable from their invasive counterparts.

**Fig. 6:**
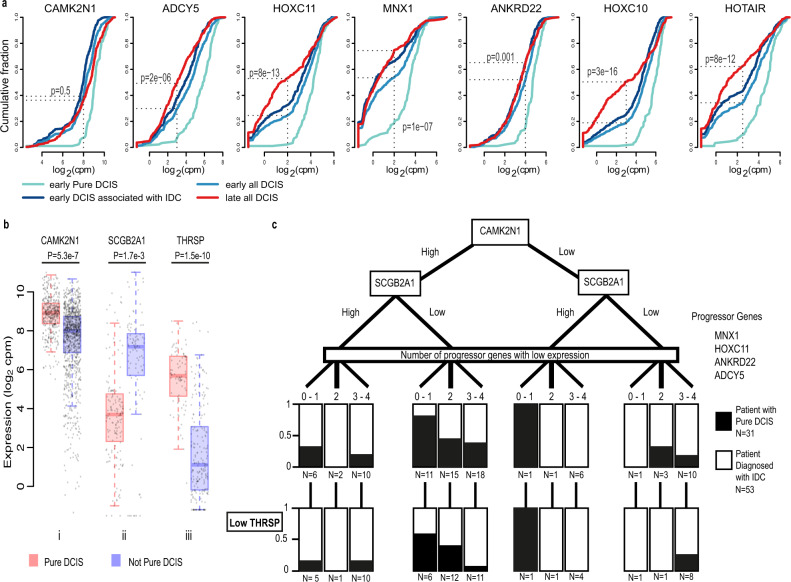
Genes displaying potential as indicators of progression from DCIS to IDC. **a** Cumulative frequency plots for differential genes between early positioned Pure DCIS and early positioned Not Pure DCIS. X axis shows the gene expression in log_2_ counts per million (CPM), Y axis shows the cumulative fraction of samples with the corresponding expression value or lower. Significance values reflect the two-sided Fisher’s exact test for a difference between cumulative fraction of all early DCIS compared to all late DCIS. **b** Expression in log_2_ counts per million (CPM), of i *CAMK2N1* for all DCIS samples (n = 385 Pure DCIS samples and n = 1014 Not Pure DCIS samples), ii of *SCGB2A1* for all samples belonging to patients in the Low Hazard group – 1 progressor gene down regulated and *CAMK2N1* high (n = 148 Pure DCIS samples and n = 97 Not Pure DCIS samples), and iii *THRSP* for all samples belonging to patients with 3–4 progressor genes down regulated, *CAMK2N1* high and *SCGB2A1* low (*n* = 84 Pure DCIS samples and n = 199 Not Pure DCIS samples). Centre line represents the median, box limits represent upper and lower quartiles, whiskers represent minimum and maximum values and at most 1.5x the interquartile range. Each point represents a sample. Differential expression analysis was done using limma-voom and two-sided *p*-values were adjusted for multiple testing using Benjamini–Hochberg correction. **c** Separation of patients with no IDC identified in our tissue biopsy. In all, 31 patients were never diagnosed with IDC after 10+ years of care, 53 patients were diagnosed with IDC in a secondary biopsy or at a later timepoint. Black/white regions reflect the proportion of patients with each diagnosis (Pure DCIS vs with IDC) within each node. Boxes in the low *THRSP* layer reflect the number of *THRSP* low patients from the node above.

To search for potential markers that could distinguish patients who would be more or less likely to progress to IDC, we first looked at the early region of the PCP. We compared samples from those patients with Pure DCIS to those patients who were diagnosed with IDC – NOT Pure DCIS (concurrent, or at any timepoint after the biopsy was taken as noted in their clinical follow up). We identified DEGs where the DCIS samples associated with an IDC diagnosis had a bimodal or skewed distribution of expression values, and the samples from Pure DCIS patients had an oppositely skewed pattern. We identified 7 such genes: *CAMK2N1*, *MNX1*, *HOXC10*, *HOXC11*, *ADCY5*, *ANKRD22* and *HOTAIR*. All showed a distribution of expression values that were lower in the NOT Pure DCIS samples as compared to Pure DCIS (Fig. 6a). If these genes were early indicators of progression potential, one might imagine that their expression changes would be enriched among all DCIS samples as they became more similar to IDC along the PCP continuum. We therefore compared the distribution of expression values in all DCIS samples from the early part of the PCP (region E1 to E2, Fig. 3b) to all DCIS samples from late in the PCP (region L1 to L2). To differing degrees, all except *CAMK2N1* showed a general decrease in the distribution of expression values in later stage samples (as defined by the PCP, Fig. 6a).

Differences in the distribution of expression values for *CAMK2N1* were exclusively linked to patient status (Pure DCIS versus Not Pure DCIS). Its expression remained discriminatory in all stages of the PCP, though it did not reach significance in later stages. This gene encodes a recently identified inhibitor of Calcium/calmodulin-stimulated protein kinase II, a protein thought to be involved in various cellular processes including cell proliferation, mammary gland lumen formation, and cancer cell metastasis. Expression of this protein kinase, encoded by *CAMK2*, is also predictive of poor breast cancer patient prognosis^44^. *CAMK2N1* itself has been reported as a prognostic marker for ovarian cancer^45^ and expression of which plays a tumour suppressive role in prostate cancer^46^ and glioma^47^, and in comparing all Pure DCIS with all other DCIS samples, is significantly down regulated in Not Pure DCIS samples (Fig. 6b).

*HOXC11*, *HOXC10* and *MNX1* each contain a homeobox domain, and *HOTAIR* is an antisense RNA whose source locus is found within a cluster of *HOXC* genes, between *HOXC11* and *HOXC12*. Homeodomain proteins function as transcription factors, regulating gene expression and cell differentiation during development, and have been frequently associated with cancer progression, where they are either up or down regulated, depending on the Hox family member and cancer type. A recent study modelling the growth expansion of DCIS posited an initial rapid expansion phase, followed by a long-term steady phase were cells were predicted to be in a cell density induced quiescent state^48^. Notably, down regulation of *HOXC10*, *HOXC11* or *MNX1* has been reported to reduce cell proliferation in a variety of different cancers^49–52^, so could suggest a possible quiescent state prior to invasion. Similarly, knockdown of the ankyrin repeat domain 22 gene, *ANKRD22*, inhibited the proliferation, invasion and epithelial-to-mesenchymal transition of breast cancer cells^53^, and a number of studies have reported high levels of expression being associated with poor outcome in non-small cell lung cancer^54^ and prostate cancer^55^, an inverse correlation to what we observe here with a ductal in situ disease. The adenylate cyclase 5 gene, *ADCY5*, is thought to be regulated by the expression of *FOXP1*, a tumour suppressor. Knockdown of *FOXP1* was followed by a significant upregulation of genes attributed to chemokine signalling pathways, including *ADCY5* ^56^.

*HOTAIR* has previously been identified as a segregation marker between two clusters of DCIS^57^, however this prior study noted that an upregulation of *HOTAIR* was associated with a more ‘aggressive’ cluster of DCIS. This aggressive cluster however, was predominantly triple-negative disease, whereas our groups were not segregated by subtype, and the DCIS samples in the latter part of our PCP were predominantly not triple negative. Other studies have reported an upregulation of *HOTAIR* when comparing human cancers to adjacent non-cancerous tissue^58^, and we also found that this lncRNA showed lower expression in our normal epithelium samples, albeit at levels similar to what we see in the DCIS associated with IDC samples from the early region of the PCP.

As *HOXC10*, *HOXC11* and *HOTAIR* loci are closely linked on the same chromosome, it seemed possible that the changes in expression that we observe could have resulted from copy number loss; however, we do not see a similarly reduced expression for *HOXC12* or *HOXC8*, the two adjacent genes.

To provide a foundation for future validation studies, we wondered whether we could use any combination of these markers to associate patients from this study, with the presence of IDC. We formulated a decision tree, focusing on protein-coding genes which may be more routinely evaluable clinically. Because of its ability to segregate the samples from the Pure DCIS group from the Not Pure DCIS group in all PCP groups, we placed *CAMK2N1* at the top of the tree, separating high and low expression categories. We then explored different ways of using information on the expression of *MNX1*, *HOXC11*, *ANKRD22* and *ADCY5*, as in no scenario did *HOXC10* seem to add additional discriminatory power. We found that simply tallying the number of these genes, hereafter termed Progressor genes, that were down-regulated enabled us to bin patients into groups within the decision tree. These 4 markers, plus *CAMK2N1*, enriched for patients who did not progress to IDC by 3.6-fold using the criteria of 0–1 gene down regulated and *CAMK2N1* high (Lower Hazard group) (36% vs 10%, - patients from the Pure DCIS group vs patients with an IDC diagnosis), whereas 3–4 genes down regulated or *CAMK2N1* low (Higher Hazard group) enriched for patients that received a diagnosis of IDC by 1.7-Fold (71% vs 42% – patients with an IDC diagnosis vs patients from the Pure DCIS group; Fig. S8). This difference within the Higher Hazard category, may suggest that many more patients might have progressed to IDC had they not been treated. This percentage is consistent with current research suggesting that between 13–53% of patients with untreated DCIS will progress to invasive disease^59^. Interestingly, we noticed that the majority of Her2-positive patients in the Pure DCIS group fell into the Low Hazard group (6 out of 8); however, we did not see this enrichment in the Not Pure DCIS group. Previous studies have noted a higher proportion of Her2-positive DCIS cases compared with that seen in invasive disease, and it has previously been suggested that a Her2 DCIS may actually be less likely to progress to IDC^60^, our findings here would support this hypothesis. We next sought to identify additional markers that could segregate the Lower Hazard group, further differentiating those patients with Pure DCIS from those diagnosed with IDC. We found *SCGB2A1*, encoding Mammaglobin B, to be significantly differential between the two groups and able to provide further discrimination between patients (Fig. 6b). High expression of *SCGB2A1* was frequently associated with high expression of *SCGB2A2* and *SCGB1D2*, encoding Mammaglobin A and lipophilin B. Expression differences at both the RNA and protein level of SCGB1D2 have also been observed in a prior study of 24 patients, comparing DCIS with and without progression to IDC^5^. All three proteins are members of the secretoglobin superfamily and are known to be upregulated in breast cancer, with SCGB2A2 and SCGB1D2 forming a multiprotein complex^61^. Using this additional marker, we were able to place 29% of Pure DCIS patients into the Lower Hazard group whereas just 3% of those with IDC fell into the Lower Hazard group (Table S2 shows expression values for high and low expression of each gene). Taking the subset of patients, where we found only DCIS in our tissue biopsy (DCIS with IDC patients and Pure DCIS patients), and blinded by any diagnosis of IDC from other tissue biopsies from the same patient, we were also able to discriminate those who had been diagnosed with IDC using our markers (Fig. 6c).

We next sought to understand why some patients with Pure DCIS, while being grouped into the Higher Hazard category, according to our marker set, had however, not been diagnosed with IDC. For this we first compared all patients high for *CAMK2N1* and low for *SCGB2A1*, with reduced expression of 3–4 progressor genes (*N* = 25 patients diagnosed with IDC; N = 7 patients with Pure DCIS). We found *PHGR1*, *THRSP* and *SERPINA5* to be highly differential between the two groups, with increased expression in Pure DCIS (Fig. 6b and Table S3). Although these genes were frequently co-expressed, we found *THRSP* able to best segregate the Pure DCIS patients from those patients diagnosed with IDC (Fig. 6c). We did not find this gene to be additionally informative for any other group on the decision tree. *THRSP* encodes the Spot14 (S14) protein, which regulates fatty acid synthesis in mammary epithelial cells^62^. Over expression of this protein was seen to reduce the tumour latency period in mice and increase proliferation; however, this same study showed an overwhelming reduction in lung metastasis in these same mice compared to controls or *THRSP* knockout mice. This gene, along with other genes involved in fatty acid biosynthesis, was also found to be down regulated in invasive growth compared to in situ growth in a mouse model of DCIS^63^. Similarly, upregulation of *SerpinA5* has been linked to reduced metastatic and invasion potential in both ovarian and breast cancer^64,65^. Comparing Pure DCIS with Not Pure DCIS for all samples with reduced expression for 3–4 progressor genes we found a number of DEGs (Table S3) previously associated with invasion and metastatic potential that were expressed at consistent levels (correlating with reduced metastasis) for all Pure DCIS samples, including *SERPINE2* and *SLPI*, both genes found to influence metastasis and contribute to vascular mimicry in a mouse model of breast cancer^66^. These Pure DCIS samples were also predominantly located in the later stage of our PCP (L1–L2 region), suggesting they may lie at the point when they need to acquire the capacity to leave the duct as the next step in their progression.

The functional diversity of these markers may indicate that multiple factors must come together for DCIS to progress to invasive breast cancer. Although we have proposed possible progression markers that will require more extensive validation, it still remains to be seen whether, and how, each of these may play a role in this disease. A recent study^7^ also looking at potential biomarkers of DCIS progression, identified the genes *FGF2*, *GAS1*, and *SFRP1* as being markers of in situ progression, suggesting their downregulation contributed to the invasiveness of epithelial cells. In support of this we also see that these 3 genes are notably down regulated as samples are arrayed along the PCP continuum. These previously described genes, although discriminatory between early and late PCP DCIS samples, were not differential between Pure DCIS patients and DCIS patients diagnosed with IDC, at any stage along the PCP.

## Discussion

Though widespread screening for breast cancer has detected disease in many more women at an early stage, a corresponding decrease in breast cancer deaths has not been forthcoming^67^. Instead, many more women are receiving treatment for non-invasive disease, which may include chemo- or radiotherapy, coupled with breast-conserving surgery or mastectomy. Numerous studies indicate that a substantial fraction of women with a diagnosis of DCIS would never progress to life-threatening invasive disease^68,69^. Therefore, many women are perhaps being overtreated using therapies with significant and long-term deleterious side effects. This realisation provokes an urgent call for a better understanding for the development of DCIS and ways to discriminate those who will progress to invasive disease, and thus require more aggressive treatment, from those who are unlikely to do so and who may opt for less extensive interventions. Our transcriptomic analysis of this large data set has enabled us to identify processes that may characterise the progression of DCIS from initiation to invasive disease and to identify candidate biomarkers, which may be associated with progression. It must be acknowledged however, that there is substantial intra- and inter-patient heterogeneity, and that not every patient displayed the same trajectory within the snap-shot in time that we analysed. However, when samples were grouped based on position on the principle curve we actually saw a surprising amount of consistency in gene expression (as a whole) across different patients. By using the format we have presented, it has been possible to gain an increase in statistical power that may not have been possible if all samples were grouped together or if a smaller number of patients were used. Though the markers we have identified will need to be validated in independent and larger cohorts, the generation of these hypotheses illustrates the utility of this large-scale dataset for the broader community.

## Methods

Participants were recruited from clinics at Duke University Medical Center, Durham, North Carolina, USA and provided consent under protocols approved by the Duke University Medical Center Institutional Review Board. This study was performed in accordance with the ethical standards as laid down in the 1964 Declaration of Helsinki. There was no compensation to research subjects for this study.

### Statistics and reproducibility

A detailed description of sample collection is found in the Laser capture micro-dissection section. In short, the sections were annotated and up three adjacent sections were micro-dissected and used to prepare 2724 RNA-seq samples. These libraries were filtered based on library depth, alignment metrics and by removing outliers as described in the Quality assessment of RNA-seq data section. The final dataset included 2222 samples, representing 1230 distinct lesions from 143 patients, with 274 lesions present as a single sample, 902 lesions present as two samples derived from different sections, and 48 lesions present as three separate samples. The filtering criteria was determined based on the QC metrics distribution in our data. No statistical method was used to predetermine sample size. All sequencing data were processed by the same pipeline, which was blinded to sample annotations, with the exception of the outlier detection step that used tissue and patient annotations to detect outliers. Sample exclusions and the reason are listed in Supplementary Data 1. Imaging mass cytometry (IMC) was carried out on 8 other patient slides including the same marker combinations as shown in Fig. 4. An additional 5 patients using Cald1, CK14 and E-Cadherin were also imaged. The image shown represents the clearest distinctions across different stages on the PCP continuum. Other patients showed similar protein localisation, and as discussed variability for these markers, however not all tissue lesions could be paired with RNA data, nor represented as many distinct positions on the PCP in a single slide.

### Patient tissues

Freshly frozen breast tissue was analysed under a Duke University IRB approved Tissue Use Protocol Pro00059726. These biopsies were originally consented for tissue banking and study under the Duke Breast SPORE grant (Pro00014678), the DUHS Biospecimen Repository and Processing Core (BRPC) Facility protocol, the DOD TVA tissue bank (Protocol #Pro00045965), or the DOD CTRA tissue bank (Protocol #Pro00044981). Primary breast cancer specimens were collected from women with an abnormal mammogram suspicious for malignancy and undergoing a medically indicated diagnostic breast core biopsy sampling who were willing to donate cores of tissue for research. After obtaining informed consent, a diagnostic core biopsy was conducted, and additional research cores were obtained. The research cores were frozen immediately in OCT embedding compound in the vapour phase of a liquid nitrogen bath or on dry ice and held frozen at −80 °C until a definitive diagnosis was made by pathologic assessment of the diagnostic cores. At time of definitive diagnosis, H&E stained frozen section slides were prepared from the research core biopsies and compared with the results from the diagnostic cores by a pathologist with expertise in breast pathology. Tissue was stored in a locked and monitored −80 °C freezer until it was used for this study.

### Tissue preparation

Frozen tissue biopsies were sectioned under RNase clean conditions. Ten serial sections of each were taken, with two sections per slide – 6 sections (10 μM) on PEN slides and 4 sections (5 μM) on glass slides. The first and last (glass) slides were subjected to H&E staining, mounted, and annotated by an experienced pathologist. Remaining sections were mounted on PEN slides, and stored for a maximum 1 week, before H&E staining immediately prior to micro-dissection.

### H&E staining

Sections were fixed in 75% ethanol for 40 s followed by 30 s in RNase free water. Sections were then treated with Hematoxylin solution (Harris Modified, Sigma-Aldrich) for 30 s, washed in water for 30 s in three different containers, before being dipped into Blueing reagent (0.1% NH4OH, Sigma-Aldrich) for 30 s followed by Eosin solution (Sigma-Aldrich) for 10 s. Lastly sections were dehydrated in rising ethanol concentrations (70, 95 and 99.5% ethanol, 30 s each) and air dried.

### Laser capture micro-dissection

Lesions were first paired up with the pathologist annotated regions, and each lesion was identified in all tissue sections prior to dissection. IDC lesions (and occasionally DCIS lesions) were more variable in their distribution through the sections and no lesion was dissected if it was not clear that we could identify the same lesion in the neighbouring section. Tissues were cut using a drop in the tube cap- laser dissection (LCM) microscope (Leica DM6000R/CTR6500) using the Leica LMD7000 system (Leica Microsystems CMS GmbH, Wetzlar, Germany). Images were taken (and confirmed by the pathologist) and cells were dissected under ×10 or ×20 magnification, with the minimal laser power necessary. Isolated cells were collected in 9 μl of lysis buffer (for RNA-seq library preparation). The tubes were then snap frozen on dry ice (with tissue remaining in the cap) and tubes stored upside at −80 °C until further processing. Lesions were collected over 3 adjacent sections and each individual dissection corresponded to 1 RNA-seq library preparation, for example a biopsy with 3 DCIS containing ducts had 9 individually dissected regions, 9 RNA-seq preparations and represented 9 samples for expression data, which were then subject to the below described quality filtering.

### RNA sequencing preparation

Samples were processed according to manufacturer’s instructions with 15 cycles of pcr amplification using the SMARTer ultra-low RNA kit V3 (Takara Bio USA, Mountain View, CA, USA). Amplified cDNA was fragmented using the Covaris LE220 sonicator (Covaris, Woburn, MA) according to the manufacturer’s instruction to yield a target fragment size of 200 bps. The sequencing library was then prepared from fragmented cDNA using NuGEN Ovation Ultralow Multiplex System (NuGEN, San Carlos, CA, USA) with 12 cycles of PCR. Finished libraries were purified from free adaptor product using RNAClean XP beads (Beckman Coulter Genomics, Brea, CA, USA). The resulting purified libraries were quantitated using a Qubit (Thermo Fisher Scientific, Waltham, MA USA) and the Kapa library quantification kits (Roche Life Science, Indianapolis, IN USA). The size range of the libraries was confirmed by the Agilent 2100 Bioanalyzer and the Agilent 4200 TapeStation (Agilent Technologies, Palo Alto, CA, USA). An equal amount of cDNA was used to pool up to 6 samples per pool.

### RNA-seq alignment and quantification

Raw reads were aligned to the GRCh38/hg38 reference genome using STAR^70^, (v2.5.2,–alignIntronMax 200000–alignMatesGapMax 200000—chimSegmentMin 15–chimJunctionOverhangMin 15, Gencode V25 gene models). Gene counts were derived using featureCounts (v1.4.3) with default options and Illumina iGenomes Refseq annotations (corresponding to GCF_000001405.30).

### Quality assessment of RNA-seq data

We obtained 2724 initial samples for analysis after excluding failed libraries with <1 million raw reads, <15% uniquely mapping reads, or <5% of the raw reads mapping to genes.

For each tissue type (DCIS, IDC, normal epithelium, benign epithelium, atypical epithelium,) we then applied the following additional filtering. Limma-Voom^71^ was used to calculate TMM normalisation factors and convert the normalised counts to log_2_ counts per million (CPM) values. A three-step filtering procedure was employed to remove low-quality samples based on their global gene expression patterns. First, the Pearson correlation between each sample and the mean log_2_(CPM) was calculated and the worst sample was iteratively removed and the mean re-calculated, this was repeated until all remaining samples had correlation ≥0.70 (≥0.65 for IDC samples due to their increased heterogeneity) to the mean. Second, individual samples that were more correlated to the mean of all samples than to the mean derived from the patient in question were excluded. Third, the correlation between each sample and the mean log_2_(CPM) for samples from same patient, was calculated and the worst sample was iteratively removed and the mean re-calculated until all remaining samples had Pearson correlation ≥0.80 (≥0.75 for IDC samples).

The thresholds were chosen to remove only samples that were either failed or were of considerably less quality compared with other samples from the same tissue and/or patient. In addition, during further validation we noticed that this filtering procedure excluded more basal samples than any other molecular subtype and we therefore opted to use more lenient thresholds for those samples (DCIS samples predicted to be basal were filtered using the IDC thresholds, and IDC samples predicted to be basal were filtered using ≥0.60 and ≥0.70 as thresholds). In total, 414 samples were removed by the first filter, 43 by the second, and 45 by the third filter, resulting in 2222 retained samples in the final dataset, representing 1230 distinct lesions from 143 patients, with 274 lesions present as a single sample, 902 lesions present as two samples derived from different sections, and 48 lesions present as three separate samples. All samples and filtering results are listed in Supplementary Data 1.

### Molecular subtype classification

Molecular subtypes (Her2, Normal, Basal, LumA, LumB) were assigned using Absolute Intrinsic Molecular Subtyping (AIMS) (Fig. S1)^10^. The AIMS package from R Bioconductor was applied on the expression counts matrix. RNA expression levels for *ESR1*, *PGR* and *ERBB2* were established based on both triple negative samples and the natural thresholds set after clustering samples. Log_2_ CPM for each gene; *ESR1*: 6, *PGR:* 6 and *ERBB2*: 10.5.

### Patient and sample group assignment

Patients were assigned to one of four categories; Pure DCIS - where ipsilateral IDC had not been reported in the patient, neither at the time, nor in follow up appointments during more than 10 years since original diagnosis (details Supplementary Data 1), *N* = 31; DCIS+ IDC - Where the biopsy dissected featured both DCIS and IDC lesions, N = 45; DCIS with IDC - Where the biopsy dissected only featured DCIS however the patient had been diagnosed with IDC (from clinical or pathology biopsies, or at a later time), *N* = 55; IDC – where no DCIS was found in the dissected biopsy, but had been diagnosed in additional biopsies; *N* = 2. Or where no DCIS was diagnosed in any of the biopsies N = 4. Normal epithelium, benign ducts, and atypia were taken from the same biopsies as above where present in the section or from additional patients diagnosed with DCIS in other biopsies (Fig. S9 shows clustering of all samples). Samples coming from patients in categories DCIS+ IDC and DCIS with IDC are collectively grouped as ‘NOT Pure’.

### Clustering of DCIS samples

Clustering and visualisations were done in R using all DCIS samples. The Limma-Voom ‘filterByExpr’ function was used to select genes expressed in at least 5% of the samples (*n* = 19366). Raw counts were TMM-normalised and transformed into log_2_(CPM) values. To visualise the data and to reduce the variation driven by patient differences, we applied principal component analysis (PC analysis; PCA) using the ‘prcomp’ function with default settings. The number of PCs used in the subsequent clustering and UMAP steps was selected as the minimum number of PCs required to explain >30% of the total variance in the data (13 PCs). Hierarchical clustering was done using the ‘hclust’ function and the ward.D2 agglomeration method. The resulting tree was cut into five clusters, with triple negative samples forming 1 of the clusters. UMAP visualisation was done using the ‘umap’ function from the umap package with default settings except increasing the number of epochs to 500, minimum distance to 0.2 and neighbours to 100 to reduce patient-specific effects.

### UMAP visualisation of all samples

Visualisation of all samples with UMAP was done in R. The Limma-Voom ‘filterByExpr’ function was used to select genes expressed in at least one of the tissue types (n = 19661). Raw counts were TMM-normalised and transformed into log_2_(CPM) values. A PCA was constructed using the ‘prcomp’ function with default settings. UMAP visualisation was done using the ‘umap’ function from the uwot package with default settings except setting the number of PCs to the minimum number of PCs required to explain >30% of the total variance in the data (16 PCs), increasing the number of epochs to 500, minimum distance to 0.2 and neighbours to 100 to reduce patient-specific effects.

### Differential expression analysis

Differential expression analysis was done using Limma-Voom, due to its ability to handle large datasets and replicate measurements for the same sample. First, expressed genes were selected by the ‘filterByExpr’ function. Calculation of normalisation factors was carried out using the TMM method. To correct for multiple samples coming from the same patient, we used a double ‘voom’ approach, including a ‘duplicationCorrection’ step with blocking based on patient. If no patient duplication was present in the contrast, we used a standard approach with a single application of ‘voom’. Fitting was done using ‘ImFit’ (with blocking and correction applied if applicable), followed by construction and calculation of contrasts using ‘contrast.fit’ function followed by ‘eBayes’. A gene was considered to be differentially expressed if the Benjamini-Hochberg adjusted *p*-value was <0.05.

### Pseudo-time analysis

A differential expression analysis was carried out as described, between DCIS and IDC samples, taken from only those patients with data from both tissue types. This was followed by a Principle Component Analysis (PCA) using only the most significant genes (*p* < 0.00001, *n* = 53). Remaining samples for DCIS, IDC, normal, benign and atypical epithelium, were then embedded onto this PCA to reveal the major patterns in the data and avoid grouping solely based on patient. Since the different tissue types were positioned on the PCA in a biologically meaningful order, we fitted a principle curve to the data and projected the samples onto the curve to arrange them by their predicted pseudo-time order. We note that arranging them according to their position on a UMAP embedding resulted in largely the same order (see Fig. S10). Linear methods such as ordering along PC1 have previously worked well for single cell data^72^ and we did not expect to be able to describe bi- or multifurcation events. We chose a principal curve over using PC1 to order the samples due to its ability to capture the expression wave along PC2.

### Gene set enrichment analysis

The R Bioconductor package RITAN (v.1.10.0) was used for gene set enrichment analysis using the MSigDB Hallmarks database. All protein-coding genes were used as a background. Terms with FDR-adjusted p-value <1e-5 are listed. To determine enrichment across the PCP continuum, we used a sliding window of 100 samples, moving 50 samples at a time, compared to all remaining samples. For the ssGSEA we used the GSVA package^73^ from R Bioconductor with the ssgsea method.

### Cell type enrichment analysis using xCell

Cell type enrichment scores were calculated using xCell (^24^) applied to FPKM-transformed counts. All studied samples (2222) were processed together. To study the change in epithelial cell enrichment across the PCP continuum we sorted the samples in PCP order and calculated a linear regression fit using the ‘lm’ function in R. Confidence intervals of the correlation coefficient r2 and the fitted line were estimated by bootstrapping using residual resampling with 10,000 replicates.

### Imaging mass cytometry

Previously stained H&E slides (used for annotation purposes prior to LCM) were first de-stained using a combination of ethanol and acidic ethanol. H&E-stained slides were submerged in 100% xylene for up to 72 h (less time for those slides more recently stained with H&E), to ease the removal of the coverslip. Slides were rinsed twice, one minute each in fresh 100% xylene to remove any residual adhesive. Following, slides were rehydrated in fresh absolute ethanol, 3 times for 1 min each, to remove the eosin stain. Slides were then placed in 1% acid alcohol (HCl in 70% ethanol) for one minute with gentle agitation to remove Haematoxylin stain. Finally, slides were rinsed twice in water. Heat induced antigen retrieval was carried out in Tris-EDTA (pH = 9.2) for 20 min, slides were then cooled and blocked with 3% BSA (Sigma) in TBS 0.3% Triton X-100 (Sigma-Aldrich) for 1 h at RT, prior to overnight incubation (4 °C) with Lanthanide metal-labelled antibodies conjugated in-house using Fluidigm’s MaxPar’s antibody conjugation kit (Fludigm); Anti-Caldesmon – 5 μg/ml (Abcam; ab215275), Anti-Lumican – 5 μg/ml (Abcam; ab198974), Anti-cytokeratine 14 − 5 μg/ml (ab236439), Smooth Muscle Actin – 3.75 μg/ml (SMA) (Thermofisher; 14-9760-82) and E-Cadherin – 5 μg/ml (BD Biosciences; 610182). Slides were then imaged using the Hyperion Imaging Mass Cytometer (Fluidigm). Following incubation, slides were first washed twice with TBS/0.1%Tween 20 and then twice with TBS. Finally, slides were rinsed once with water and incubated with_0.5 μM Cell-ID Intercalator-Ir (Fluidigm, 201192B) at RT. After 15 min slides were briefly rinsed with water and air-dried for at least 30 min before IMC acquisition.

### IMC acquisition

IMC images were acquired using the Hyperion Imaging Mass Cytometer (Fluidigm). The air-dried slide was loaded into the imaging module, where an optical preview of the ROIs was recorded for laser ablation. Tissues were ablated by a UV-laser spot-by-spot, line-by-line at a resolution of 1 um and a frequency of 200 Hz. IMC datasets, saved by the Hyperion instrument as.mcd files, were initially converted to the *Zarr*format, preserving the entire signal dynamic range and metadata, using a custom python script available at https://github.com/IMAXT/imc-nuclear-segmentation ^74^. The resulting zarr datasets were visualised using a custom-made IMC viewer tool, also written in python, operating on a jupyter notebook instance (available at https://github.com/IMAXT/imaxt-image).

### Patient marker classifier for group assignment on the decision tree

High and low expression of each marker gene was based on the majority segregation between Pure DCIS and Not Pure DCIS. Table S2 provides additional information regarding the expression levels for each gene. A patient was placed in a group based on a minimum of two samples representing the ‘associated with IDC’ expression levels, this being low *MNX1*, low *HOXC11*, low *ANKRD22*, low *ADCY5*, High *SCGB2A1*, low *Camk2N1* and low *THRSP*. Two patients were removed from the decision trees as data was only available for 1 sample.

### Reporting summary

Further information on research design is available in the Nature Research Reporting Summary linked to this article.

## Supplementary information

Supplementary information

Supplementary Data 1

Supplementary Data 2

Reporting Summary

## Data Availability

The Raw sequencing data (aligned to GRch38/hg38) generated in this study have been deposited in the European Genome-Phenome Archive under the Dataset ID number EGAD00001008586. The data generated from these patient samples are available under restricted access. It is stated in the patient consent forms for the tissue collection that any future research on samples or data must first be approved by a Data Access Committee (DAC). Uploaded sequencing data, and IMC data displayed within this publication, is therefore available on application to the Data Access Committee upon request to clare.rebbeck@cruk.cam.ac.uk. Data is available to the scientific community with the condition that anonymity is maintained. The results generated from the comparative analyses supporting the findings of this study are available within the paper and its supplementary information/ data files. Gene set enrichment analysis used the MSigDB Hallmarks database from R/Bioconductor RITANdata (v1.10.0).

## Code availability

Mcd files generated from IMC, were converted to the *Zarr*format, using a custom python script available at https://github.com/IMAXT/imc-nuclear-segmentation ^74^.The resulting zarr datasets were visualised using a custom-made IMC viewer tool, also written in python, operating on a jupyter notebook instance (available at https://github.com/IMAXT/imaxt-image).
