# Supplementary material for: Gene expression signatures of individual ductal carcinoma in situ lesions identify processes and biomarkers associated with progression towards invasive ductal carcinoma
Source: Nat Commun. 2022 Jun 13;13:3399. doi: 10.1038/s41467-022-30573-4 (PMC9192778; doi:10.1038/s41467-022-30573-4)
Supplement: Supplementary file 1 — Supplementary information [file 41467_2022_30573_MOESM1_ESM.pdf]

Supplementary Information

Gene	Fold Change All	Adj.P value	Fold Change very early	Adj.P value
GLTSCR2	1.67	2.8e-69	0.9	1.6e-14
RPL5	1.42	1.3e-66	0.8	2.1e-16
RPS6	1.39	1.0e-57	0.8	2.9e-14
RPL13	1.4	6.5e-47	0.8	3.0e-12
RPL34	1.3	2.5e-46	0.8	6.6e-12
EIF3L	1.3	2.6e-45	0.8	1.4e-14
RPS9	1.3	3.6e-45	0.7	7.0e-10
RPS4X	1.1	1.9e-44	0.6	1.1e-10
RPL11	1.1	3.0e-43	0.7	10.0e-12
RPL26	1.3	8.5e-41	0.7	7.0e-11
EEF1G	1.1	2.4e-37	0.8	6.8e-14
FBL	1.2	2.6e-37	0.8	2.0e-12
RPLP2	0.9	2.4e-36	0.7	2.7e-12
TPT1	0.9	2e-35	0.5	7.2e-10
GNB2L1	1.0	2.1e-35	0.6	9.9e-10
RPL15	0.9	5.3e-35	0.7	4.2e-15
FAU	0.9	9.9e-35	0.6	1.2e-10
RPL14	1.0	2.8e-34	0.6	7.1e-10
RPS25	1.4	3.2e-33	0.8	7.1e-10

Table S1 Ribosomal biogenesis genes significantly down regulated in DCIS compared to normal tissue.

All – refers to analysis comparing all normal/benign tissues with Pure DCIS

Very early – refers to analysis comparing normal tissues with DCIS tissues in the very early part of the PCP continuum.

Gene list represents the cluster of highly significant genes that were shared between All analysis and Very early analysis. Differential expression analysis was done using limma-voom and two-sided p-values were adjusted for multiple testing using Benjamini-Hochberg correction.

REFSEQ gene ID	Low expression < log2CPM > High expression
CAMK2N1	< 7 >
MNX1	< 3 >
HOXC11	< 3 >
ANKRD22	< 3 >
ADCY5	< 2.5 >
SCGB2A1	< 5 >
THRSP	< 3 >

Table S2 Gene expression thresholds.
Distinction for high and low expression for each gene used in the classification (in log_2 counts per million (CPM)).

	CAMK2N1 + / SCGB2A1 - / 3-4 progressor genes down				All 3-4 progressor genes down			
			Mean Expression				Mean Expression	
**REFSEQ gene ID**	log2FC	Adj.PValue	Pure DCIS	Not Pure DCIS	log2FC	Adj.PValue	Pure DCIS	Not Pure DCIS
PHGR1	4.33	8.4e-13	7.88	4.00	4.07	3.05e-22	6.93	3.93
THRSP	4.04	1.5e-10	5.54	1.69	1.5	0.01	3.86	2.11
SERPINA5	2.48	1e-8	7.00	4.74	2.8	7.24e-19	6.86	4.48
LYPD6B	1.36	0.01	6.4	5.11	2.45	3.28e-17	6.36	4.01
GFRA1	1.88	9.3e-4	9.25	7.38	2.97	6.9e-17	9.12	5.99
NPNT	1.6	0.004	8.12	6.15	2.04	5.4e-10	7.85	5.38
SLPI	2.01	0.25	2.18	4.01	2.7	0.03	2.32	4.99
SERPINE2	2.31	0.14	1.39	3.11	2.46	0.01	1.57	3.49
FBLN2	2.22	0.1	1.11	2.92	2.43	0.006	1.23	3.23
MSL3P1	1.51	0.27	0.05	1.4	2.17	0.004	0	1.95

Table S3 Differential genes in the High Hazard group.
Genes distinguishing Pure DCIS from DCIS associated with IDC (Not Pure DCIS) in the Higher Hazard group of patients. Differential genes are from analysis first using only patients with *CAMK2N1* high / *SCGB2A1* low and reduced expression of 3-4 progressor genes, and then second using all patients with reduced expression of 3-4 progressor genes, regardless of *CAMK2N1* or *SCGB2A1* expression. Differential expression analysis was done using limma-voom and two-sided p-values were adjusted for multiple testing using Benjamini-Hochberg correction.

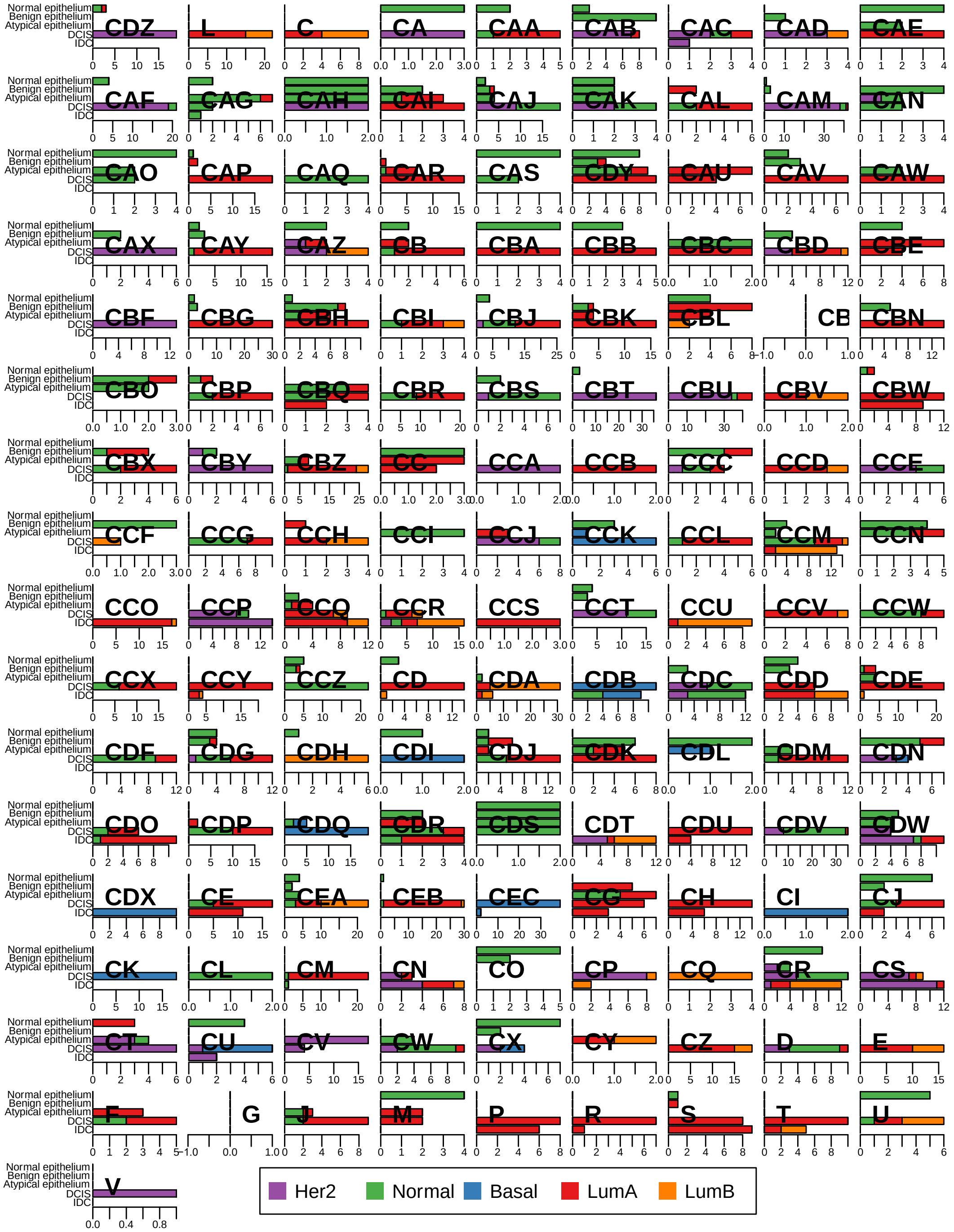

Figure S1. Patient subtype classification.

Number of samples (after filtering) assigned with each AIMS subtype classification, from each patient. We found 52% of patients had mixed AIMS classifications for their DCIS samples, and 46% having mixed classifications for their IDC samples.

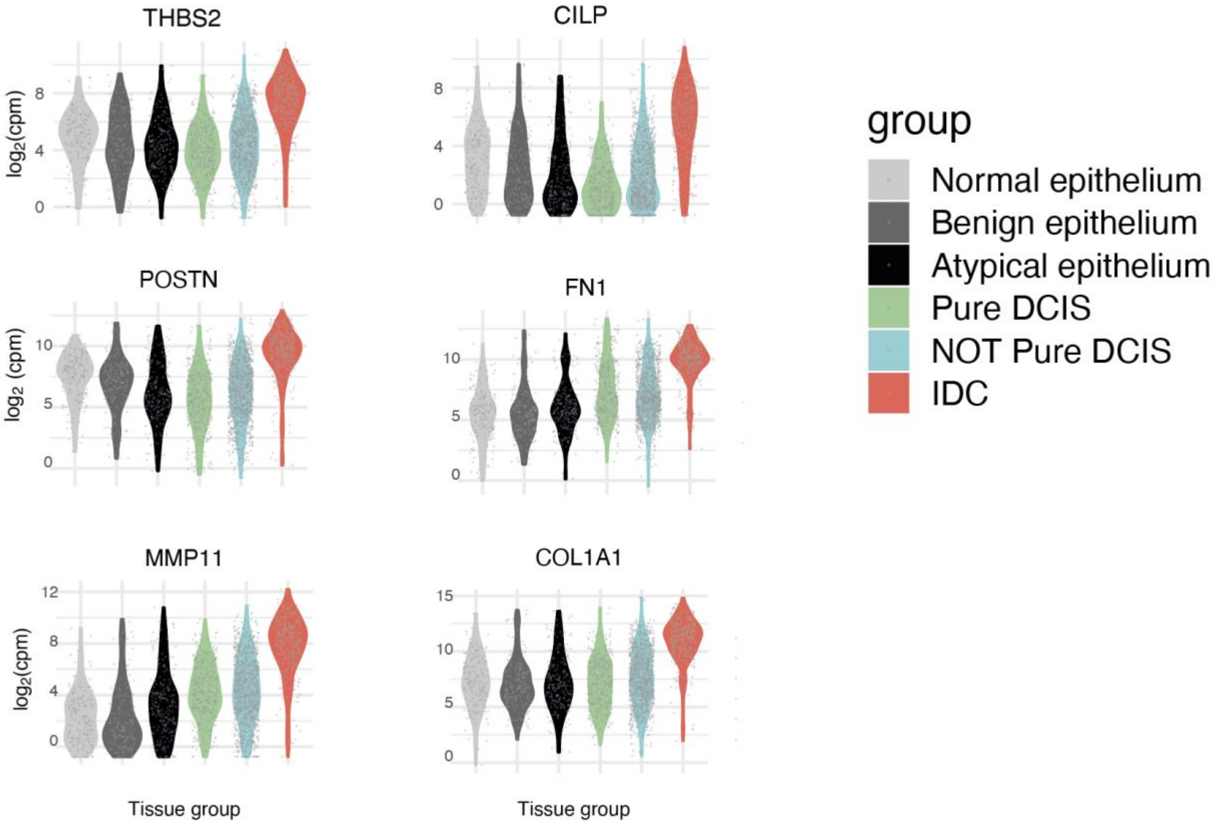

Figure S2. Differentially expressed genes between DCIS and co-occurring IDC.
Expression distribution for example genes that showed a progressive shift among different tissue groups. Each sample is represented by a grey dot and a kernel density plot is overlaid.

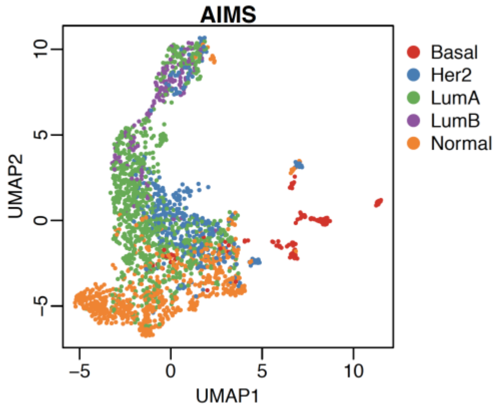

Figure S3. UMAP visualization using the same 53 genes that were used to construct the PCA plot in Fig. 3a. UMAP separates the samples more strongly by subtype compared with PCA and most triple-negative (basal) samples cluster separately.

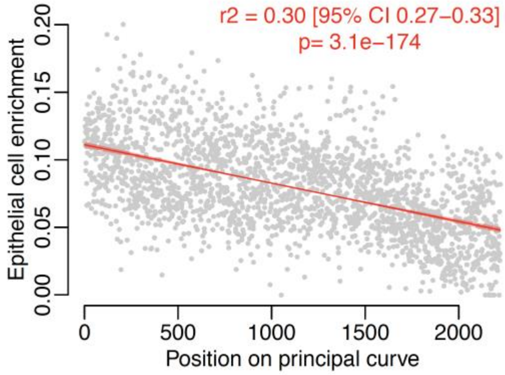

Figure S4. Epithelial cell enrichment calculated using xCell.
All samples are sorted in the order determined by the principal curve projection (PCP). The line indicates the linear regression fit with a 95% confidence interval and two-sided p-value of association.

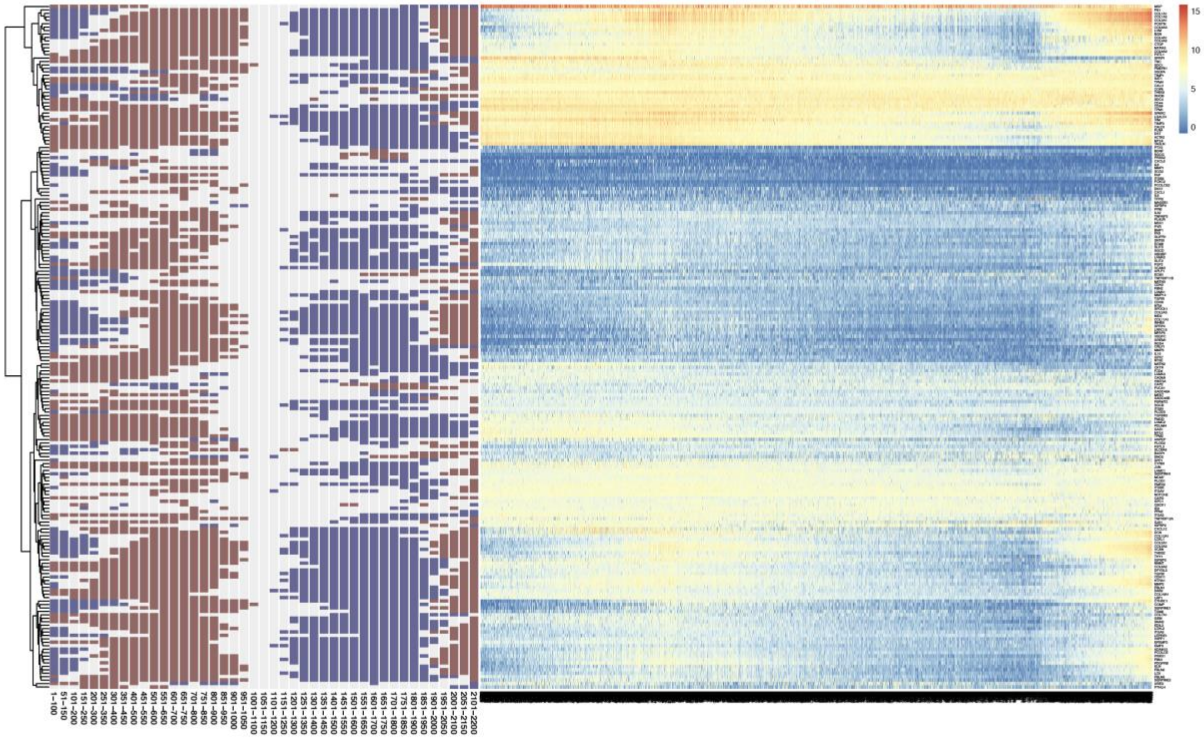

Figure S5. Epithelial to Mesenchymal transition occurs twice in the PCP continuum

Heatmap showing relative gene expression for genes listed within the Epithelial to Mesenchymal transition Hallmark signature. Samples were ordered according to the principal curve projection. Bars to the left of the heat map reflect the differential expression analysis between the 100 samples in that block against all other samples. Genes that were significantly up- (red) or down-regulated (blue) are highlighted. The threshold for being red or blue was p.adj.<0.05. Differential expression analysis was done using limma-voom and two-sided p-values were adjusted for multiple testing using Benjamini-Hochberg correction.

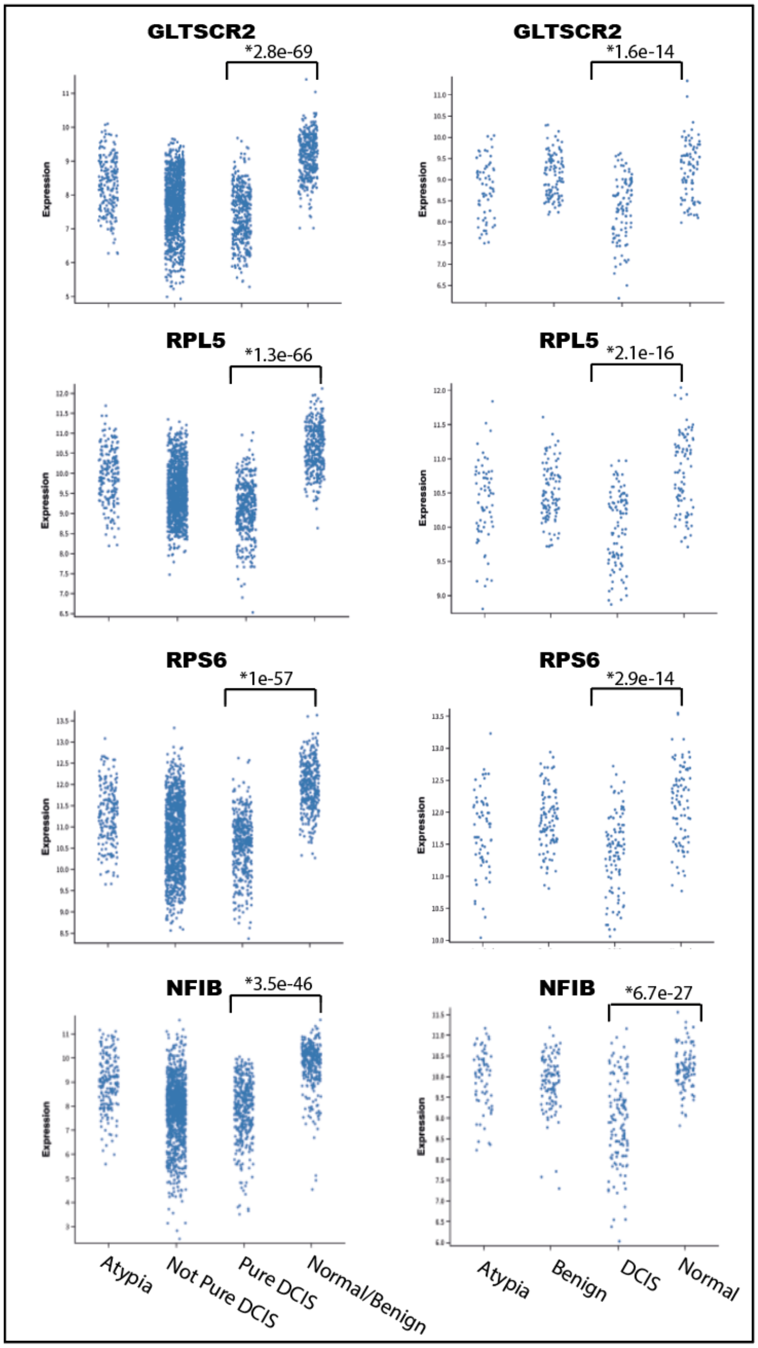

Figure S6. Differentially Expressed genes found when comparing normal/ benign ductal tissue to DCIS samples.

Expression distribution of samples in Log_2 counts per million (CPM). Left panel show all samples with each tissue type, right panel show samples in the very early region of the PCP continuum. * Indicates Adj. P value. Differential expression analysis was done using limma-voom and two-sided p-values were adjusted for multiple testing using Benjamini-Hochberg correction.

A

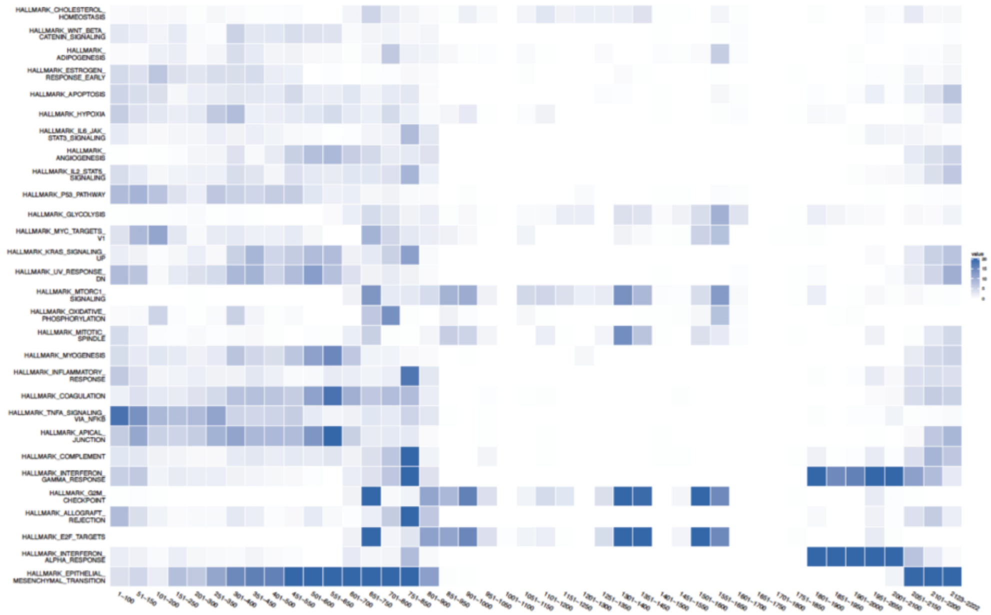
HALLMARK_CHOLESTEROL_HOMEOSTASIS
HALLMARK_WNT_BETA_CATENIN_SIGNALING
HALLMARK_ADIPOGENESIS
HALLMARK_ESTROGEN_RESPONSE_EARLY
HALLMARK_APOPTOSIS
HALLMARK_HYPOXIA
HALLMARK_IL6_JAK_STAT3_SIGNALING
HALLMARK_ANGIOGENESIS
HALLMARK_IL2_STAT5_SIGNALING
HALLMARK_P53_PATHWAY
HALLMARK_GLYCOLYSIS
HALLMARK_MYC_TARGETS_V1
HALLMARK_KRAS_SIGNALING_UP
HALLMARK_UV_RESPONSE_DN
HALLMARK_MTORC1_SIGNALING
HALLMARK_OXIDATIVE_PHOSPHORYLATION
HALLMARK_MITOTIC_SPINDLE
HALLMARK_MYOGENESIS
HALLMARK_INFLAMMATORY_RESPONSE
HALLMARK_COAGULATION
HALLMARK_TNFA_SIGNALING_VIA_NFKB
HALLMARK_APICAL_JUNCTION
HALLMARK_COMPLEMENT
HALLMARK_INTERFERON_GAMMA_RESPONSE
HALLMARK_G2M_CHECKPOINT
HALLMARK_ALLOGRAFT_REJECTION
HALLMARK_E2F_TARGETS
HALLMARK_INTERFERON_ALPHA_RESPONSE
HALLMARK_EPITHELIAL_MESENCHYMAL_TRANSITION

B

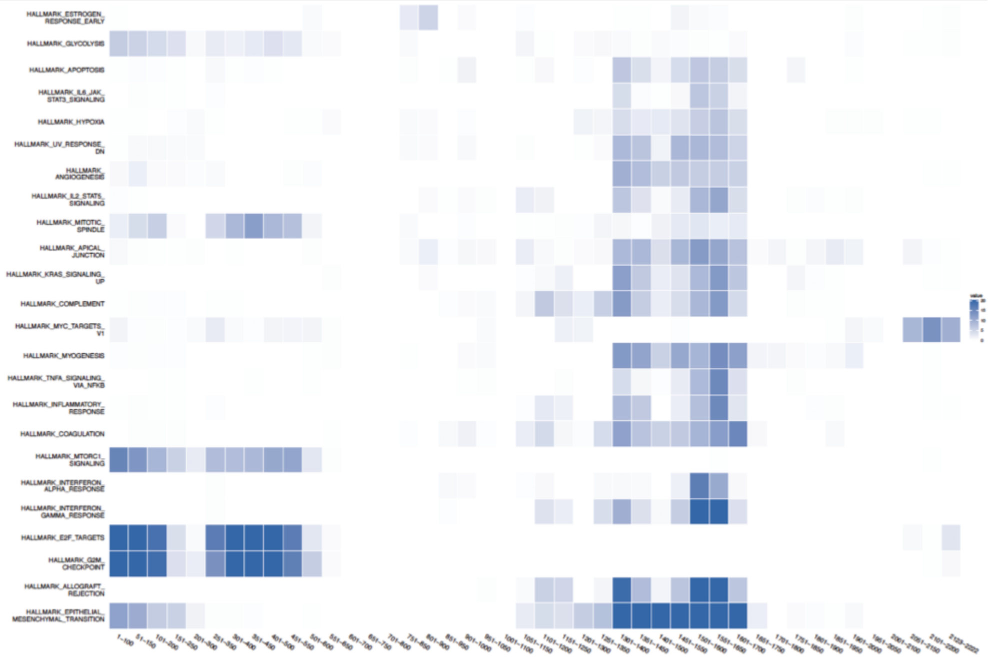
HALLMARK_ESTROGEN_RESPONSE_EARLY
HALLMARK_GLYCOLYSIS
HALLMARK_APOPTOSIS
HALLMARK_IL6_JAK_STAT3_SIGNALING
HALLMARK_HYPOXIA
HALLMARK_UV_RESPONSE_DN
HALLMARK_ANGIOGENESIS
HALLMARK_IL2_STAT5_SIGNALING
HALLMARK_MITOTIC_SPINDLE
HALLMARK_APICAL_JUNCTION
HALLMARK_KRAS_SIGNALING_UP
HALLMARK_COMPLEMENT
HALLMARK_MYC_TARGETS_V1
HALLMARK_MYOGENESIS
HALLMARK_TNFA_SIGNALING_VIA_NFKB
HALLMARK_INFLAMMATORY_RESPONSE
HALLMARK_COAGULATION
HALLMARK_MTORC1_SIGNALING
HALLMARK_INTERFERON_ALPHA_RESPONSE
HALLMARK_INTERFERON_GAMMA_RESPONSE
HALLMARK_E2F_TARGETS
HALLMARK_G2M_CHECKPOINT
HALLMARK_ALLOGRAFT_REJECTION
HALLMARK_EPITHELIAL_MESENCHYMAL_TRANSITION

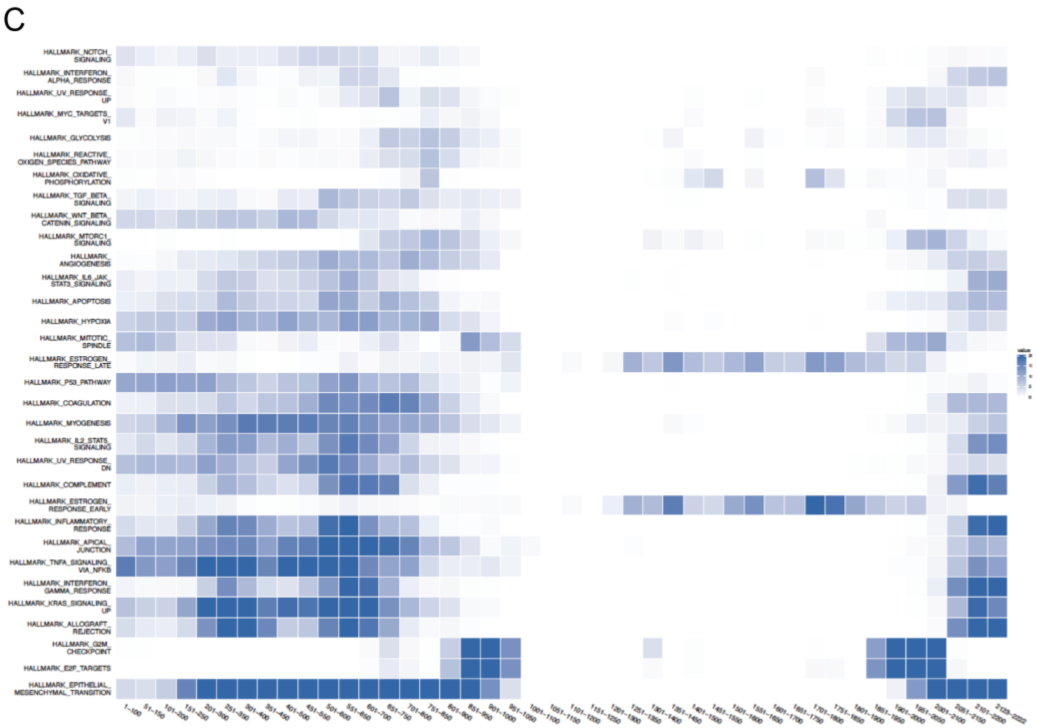

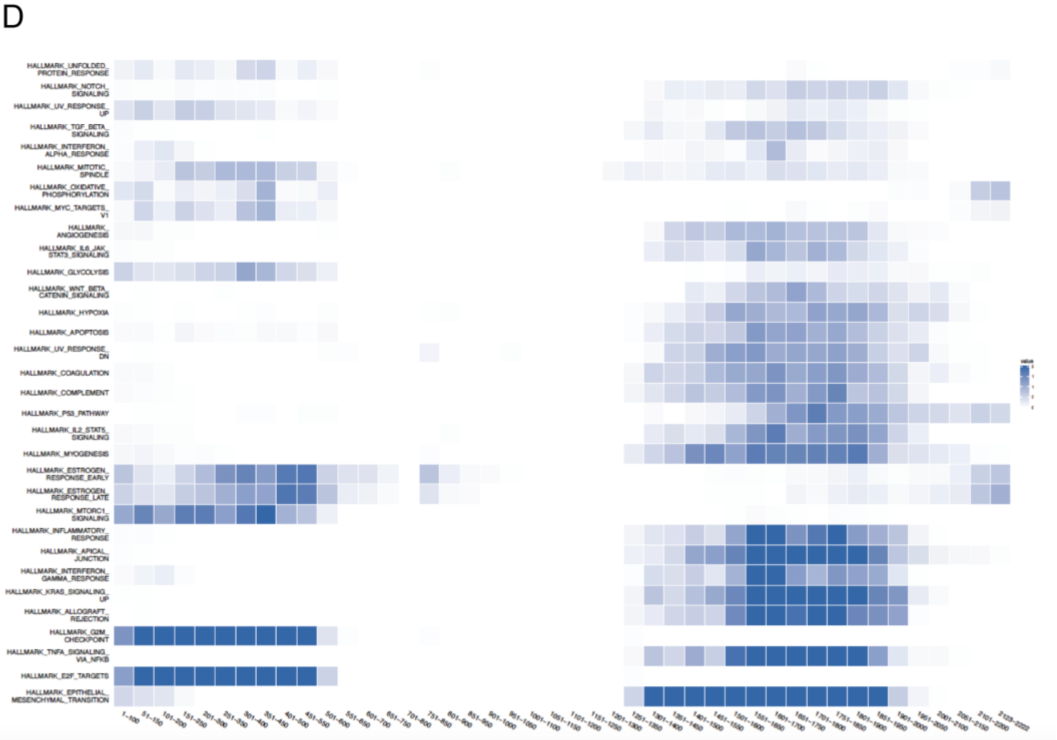

Figure S7. MSigDB Hallmark signatures up along the PCP continuum

Up (**A**) and down (**B**) for all ER positive samples. Up (**C**) and down (**D**) for all ER negative. Samples in order of PCP. Hallmark signature enrichment was calculated for samples within each bin compared to all remaining samples.

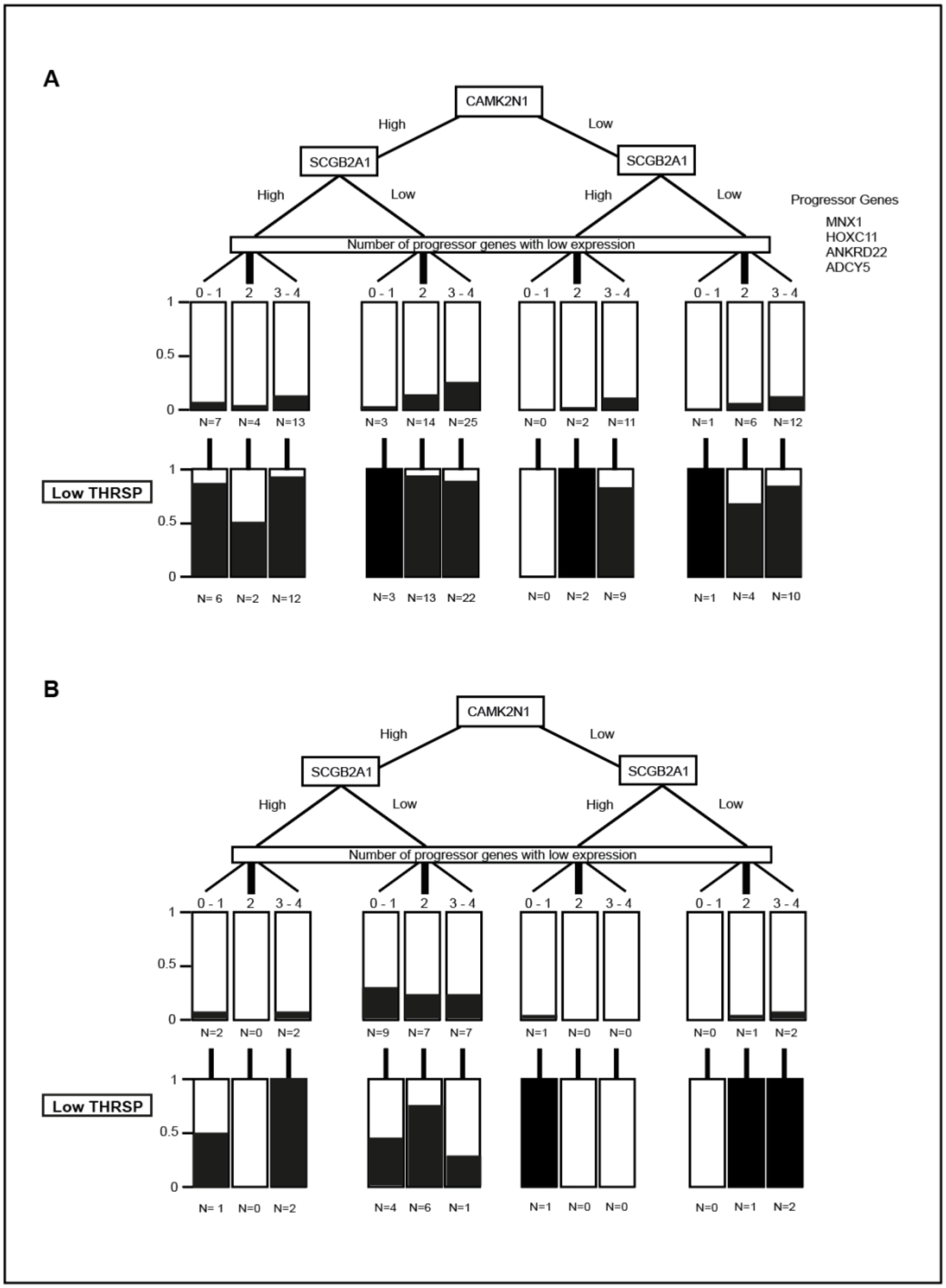

Figure S8. Decision tree for all samples.

Separation of all patients (**A**) diagnosed with IDC, N = 98 [Two patients were removed from the decision trees as data was only available for 1 sample], and (**B**) that were never diagnosed with IDC (Pure DCIS), N = 31. Black bars represent the proportion of the total that fall in that node, e.g. N = 2 is 2% where the total is 98 or 6.4% where the total is 31. Boxes in the low *THRSP* layer represent the proportion of the group above.

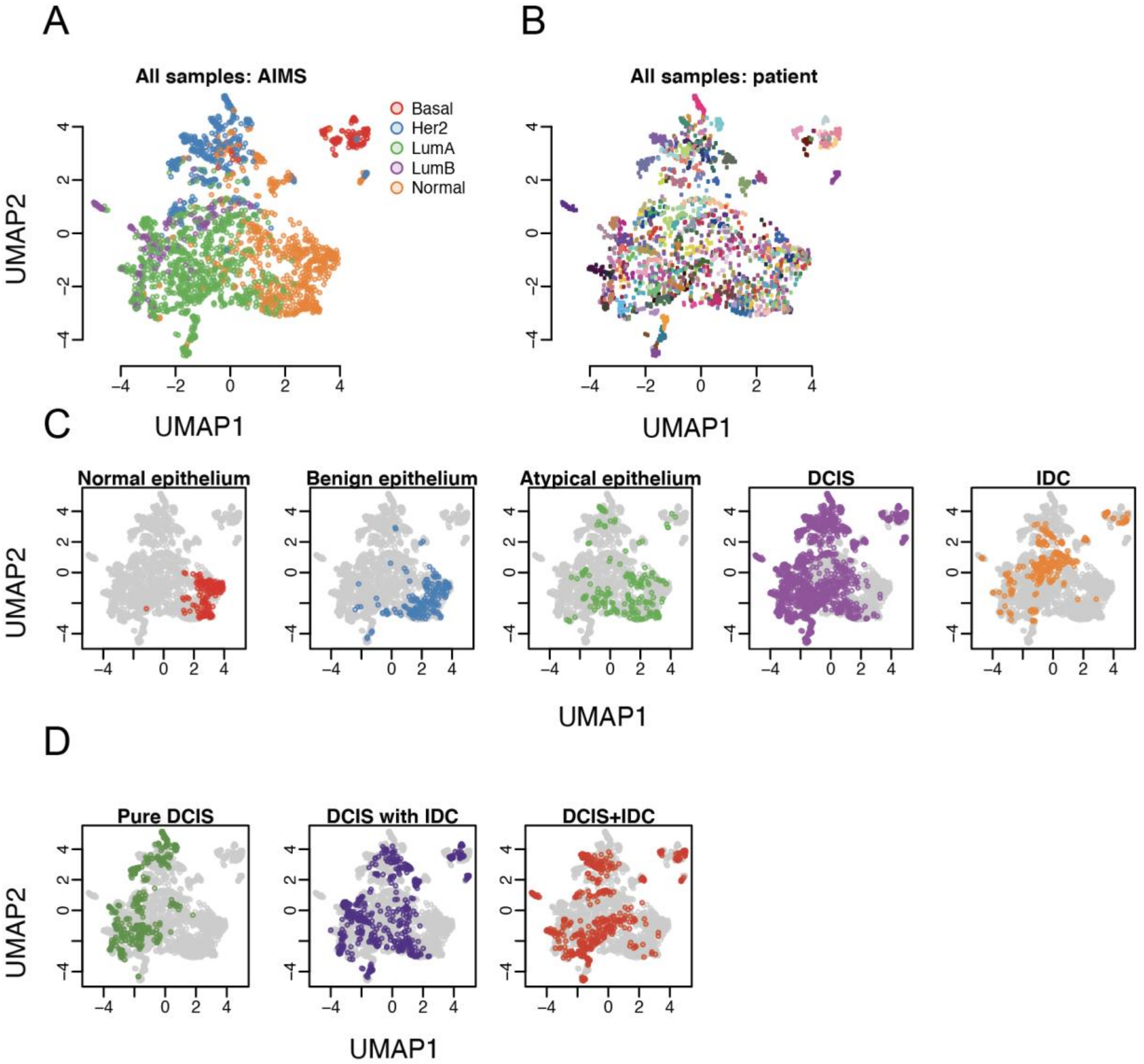

Figure S9. Sample clustering.

Principal component analysis (PCA) followed by uniform manifold approximation and projection (UMAP) for all samples that passed quality filters. (**A**) All samples coloured by their AIMS subtype classification. (**B**) All samples coloured by which patient they came from. (**C**) Distribution of each tissue type – coloured - against all samples – grey. (**D**) Distribution of each DCIS group – coloured – against all samples – grey.

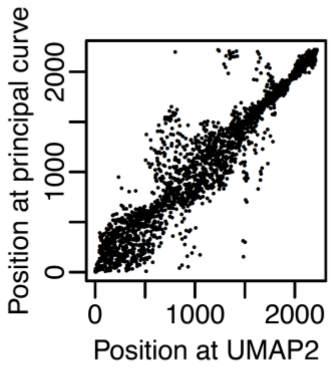

Figure S10. Ranking of samples across UMAP2 and across the principal curve, using the same 53 genes used to construct the PCA plot in Fig. 3a.
