# Supplementary material for: Gene expression signatures of individual ductal carcinoma in situ lesions identify processes and biomarkers associated with progression towards invasive ductal carcinoma
Source: Nat Commun. 2022 Jun 13;13:3399. doi: 10.1038/s41467-022-30573-4 (PMC9192778; doi:10.1038/s41467-022-30573-4)
Supplement: Supplementary file 4 — Reporting Summary [file 41467_2022_30573_MOESM4_ESM.pdf]

nature portfolio

Corresponding author(s): Clare Rebbeck, Gregory Hannon and Herbert Lylery

Last updated by author(s): 22-04-14

Reporting Summary

Nature Portfolio wishes to improve the reproducibility of the work that we publish. This form provides structure for consistency and transparency in reporting. For further information on Nature Portfolio policies, see our Editorial Policies and the Editorial Policy Checklist.

Statistics

For all statistical analyses, confirm that the following items are present in the figure legend, table legend, main text, or Methods section.

n/a	Confirmed	
☐	☒	The exact sample size (*n*) for each experimental group/condition, given as a discrete number and unit of measurement
☐	☒	A statement on whether measurements were taken from distinct samples or whether the same sample was measured repeatedly
☐	☒	The statistical test(s) used AND whether they are one- or two-sided<br>*Only common tests should be described solely by name; describe more complex techniques in the Methods section.*
☒	☐	A description of all covariates tested
☐	☒	A description of any assumptions or corrections, such as tests of normality and adjustment for multiple comparisons
☐	☒	A full description of the statistical parameters including central tendency (e.g. means) or other basic estimates (e.g. regression coefficient) AND variation (e.g. standard deviation) or associated estimates of uncertainty (e.g. confidence intervals)
☐	☒	For null hypothesis testing, the test statistic (e.g. *F*, *t*, *r*) with confidence intervals, effect sizes, degrees of freedom and *P* value noted<br>*Give P values as exact values whenever suitable.*
☒	☐	For Bayesian analysis, information on the choice of priors and Markov chain Monte Carlo settings
☒	☐	For hierarchical and complex designs, identification of the appropriate level for tests and full reporting of outcomes
☐	☒	Estimates of effect sizes (e.g. Cohen's *d*, Pearson's *r*), indicating how they were calculated

Our web collection on statistics for biologists contains articles on many of the points above.

Software and code

Policy information about availability of computer code

Data collection	No software was used for data collection
Data analysis	The bioinformatic analyses were done using a combination of publicly available software: STAR (v2.5.2), featureCounts (v1.4.3), Limma-Voom (v3.42.0 or v3.42.2), RITAN (v1.10.0), xCell (v1.1.0), and AIMS (v1.18.0). Statistical analysis and visualization of sequencing data was done in R using the hclust, prcomp, and lm functions from that stats package (v3.6.2 or v3.6.3), and the umap (v0.2.4.1 or v0.2.7.0), princurve (v2.1.5 or v2.1.6), and GSVA (v1.34.0) packages. The MSigDB Hallmarks database from RITANdata (v1.10.0) was used for gene set enrichment analysis.

For manuscripts utilizing custom algorithms or software that are central to the research but not yet described in published literature, software must be made available to editors and reviewers. We strongly encourage code deposition in a community repository (e.g. GitHub). See the Nature Portfolio guidelines for submitting code & software for further information.

Data

Policy information about availability of data

All manuscripts must include a data availability statement. This statement should provide the following information, where applicable:

- Accession codes, unique identifiers, or web links for publicly available datasets
- A description of any restrictions on data availability
- For clinical datasets or third party data, please ensure that the statement adheres to our policy

The Raw sequencing data (aligned to GRch38/hg38) generated in this study have been deposited in the European Genome-Phenome Archive under the study ID number EGAS00001005370,(https://ega-archive.org/datasets/EGAD00001008586). The data generated from these patient samples are available under restricted access. It is stated in the patient consent forms for the tissue collection that any future research on samples or data must first be approved by a Data Access

Committee (DAC). Uploaded data is therefore available on application to the Data Access Committee upon request to clare.rebbeck@cruk.cam.ac.uk. Data is available to the scientific community with the condition that anonymity is maintained. The results generated from the comparative analyses supporting the findings of this study are available within the paper and its supplementary information/ data files
Gene set enrichment analysis used the MSigDB Hallmarks database from R/Bioconductor RITANdata (v1.10.0).

Field-specific reporting

Please select the one below that is the best fit for your research. If you are not sure, read the appropriate sections before making your selection.

☒ Life sciences ☐ Behavioural & social sciences ☐ Ecological, evolutionary & environmental sciences

For a reference copy of the document with all sections, see nature.com/documents/nr-reporting-summary-flat.pdf

Life sciences study design

All studies must disclose on these points even when the disclosure is negative.

Sample size

No statistical method was used to predetermine sample size. Sample sizes were determined based on the maximum number of samples that met into the criteria being tested.

Data exclusions

In all experiments, libraries with <1 million raw reads, <15 % uniquely mapping reads, or <5 % of the raw reads mapping to genes were excluded from the study. Libraries with low correlation with other samples from the same tissue and/or patient were excluded from the analysis, as described in the methods. These exclusions could not be pre-established before generating the data. However, it was deemed necessary to exclude failed sequencing libraries in a systematic way and before performing downstream analyses. The hard thresholds essentially removed completely failed libraries. The correlation-based criteria primarily removed partially failed or low-quality libraries that performed poorly in terms of quality control metrics compared to the other libraries from the same tissue and/or patient.
One patient for whom we had DCIS and IDC annotated within our biopsy, was excluded in the experiment comparing DCIS and co-occuring IDC. The reason being in confirming LCM regions where of original annotation, the pathologist could not be sure that the annotated and dissected regions were in fact IDC. The non IDC samples from this patient were used for all other analyses.
Patients with only 1 sample for DCIS were excluded from the decision tree, as criteria was a minimum of 2 samples must fall into the designated group.

Replication

All analyses on the data are/were reproducible. For reproducibility of data collection we captured corresponding lesions from 3 adjacent serial sections. sequencing was done from 2 adjacent lesions, and where sequencing had been of poor quality in 1 of the 2, a 3rd adjacent lesion was sequenced. In some instances all 3 sections for a particular lesion were sequenced and retained after quality filtering.

Randomization

samples were randomized for sequencing. For analyses randomization was not relevant as all samples that met the question criteria were used.

Blinding

All sequencing libraries were processed by the same pipeline. The experiment to generate the fitted principle curve was blinded to tissue annotation. The experiments comparing different stages on the PPC was blinded to sample choice (groups were chosen based on a position along the PPC). Blinding for other analysis was not relevant.

Reporting for specific materials, systems and methods

We require information from authors about some types of materials, experimental systems and methods used in many studies. Here, indicate whether each material, system or method listed is relevant to your study. If you are not sure if a list item applies to your research, read the appropriate section before selecting a response.

Materials & experimental systems

n/a	Involved in the study	
☐	☒	Antibodies
☒	☐	Eukaryotic cell lines
☒	☐	Palaeontology and archaeology
☒	☐	Animals and other organisms
☐	☒	Human research participants
☒	☐	Clinical data
☒	☐	Dual use research of concern

Methods

n/a	Involved in the study	
☒	☐	ChIP-seq
☒	☐	Flow cytometry
☒	☐	MRI-based neuroimaging

Antibodies

Antibodies used

Lanthanide metal-labelled antibodies were either purchased from Fluidigm or conjugated in-house using Fluidigm's MaxPar's antibody conjugation kit (Fludigm).
Antibodies used for IMC:
anti-Lumican (EPR8898(2))- Abcam - ab198974 - 5ug/ml
anti-Caldesmon (E89)- Abcam - ab215275 - 5ug/ml

anti-cytokeratin 14 (SP53) - Abcam - ab236439 -5ug/ml
anti-E Cadherin (EP700Y) - Abcam - ab201499 - 5ug/ ml
anti - Smooth muscle actin (SMA) (1A4)- Thermofisher 14-9760-82 - 3.75ug/ml

Validation

anti-Lumican - Synthetic peptide within Human Lumican, abcam statement for recombinant antibodies"improved sensitivity and confirmed specificity" .antibody shown to stain specific cell types by abcam on paraffin embedded human skin tissue and Immunohistochemical analysis of paraffin embedded Human kidney tissue. No positive staining shown so far by other in human dcis and only detected in our own staining for some lesions. Noted by Protein atlas that protein expression did not always always correlate with rna expression - antibody staining in our own samples did in part correlate with RNA expression from our own data in that samples with high expression of Lum RNA did show staining in some of the corresponding image lesions. staining location however was consistent and appeared specific.
Anti-Caldesmon. antibody staining correlated with our own rna data, and location of protein detected by IMC matched that detected by others in the mammary duct e. g Stevenson et al PNAS 2020.
anti-cytokeratin 14. - validated by abcam - KRT14 knockout A431 cells compared with WT A431 cells, using immunohistochemistry and western blot. Also showed specific staining on frozen Rat skin sections and human prostate tissue . location of protein in our analysis also matched that found by other publications(using different antibody clones) e.g Dabbs et al. Mod. Path 2006, and the proteinatlas.org (also different antibody clones) for the mammary duct, where the protein is shown to be expressed in both glandular and myoepithlial cells lining the mammary duct of human breast tissue. samples with high RNA expression in our data were seen to have strong staining for this marker and those with low RNA expression in our data had limited or no staining for this marker in our tissue samples.
anti-E-Cadherin - Synthetic peptide within Human E-Cadherin. abcam statement for recombinant antibodies"improved sensitivity and confirmed specificity". Antibody show to mark specifically by abcam on multiple human tissue sections, including breast tissue
anti - Smooth muscle actin (SMA) - antibody validated by ThermoFisher "This Antibody was verified by Relative expression to ensure that the antibody binds to the antigen stated",and protein staining done by thermofisher on human DCIS showed similar staining patterns. Protein location supported by the literature for both mouse and human breast tissue (using a different antibody clone) , e.g Russell et al. Am J Pathol 2015 and Proteinatlas.org (using a different antibody clone) where, in human breast tissue, it is seen to mark predominantly myoepithelial and also glandular cells.

Human research participants

Policy information about studies involving human research participants

Population characteristics

Tissue was donated by women typically aged 50 plus year, however some were younger. full details of age is supplied in supplementary table 4. this table also includes diagnosis of either DCIS and/ or IDC for each patient. all samples were gathered prior to treatment.

Recruitment

Tissue was provided by Duke medical center where women agreed to donate tissues following a abnormal mammography. There would be selection bias since samples are collected from oncology clinics at Duke which may reduce sample diversity including age and socioeconomic status.

Ethics oversight

Participants were recruited from clinics at Duke University Medical Center, Durham, North Carolina, USA and provided consent under protocols approved by the Duke University Medical Center Institutional Review Board.

Note that full information on the approval of the study protocol must also be provided in the manuscript.
